# The association between body mass index and abdominal obesity with hypertension among South Asian population: findings from nationally representative surveys

**DOI:** 10.1186/s40885-023-00257-2

**Published:** 2024-02-01

**Authors:** Rajat Das Gupta, Ateeb Ahmad Parray, Rohan Jay Kothadia, Orindom Shing Pulock, Susmita Dey Pinky, Shams Shabab Haider, Maxwell Akonde, Mohammad Rifat Haider

**Affiliations:** 1 Department of Epidemiology and Biostatistics, Arnold School of Public Health, University of South Carolina, Columbia, SC 29208 USA; 2 Department of International Health, Johns Hopkins Bloomberg School of Public Health, Johns Hopkins University, 615N Wolfe St, Baltimore, MD 21205 USA; 3 Arnold School of Public Health, University of South Carolina, Columbia, SC 29208 USA; 4 Department of Medicine, Chittagong Medical College Hospital, K B Fazlul Kader Road, Panchlaish, Chattogram, 4203 Bangladesh; 5 Department of Cardiology, Hammersmith Hospital, Imperial College NHS Healthcare Trust, London, W12 0HS UK; 6 Johns Hopkins Bloomberg School of Public Health, 615 N Wolfe St., Baltimore, MD 21205 USA; 7 Department of Health Policy and Management, College of Public Health, University of Georgia, Wright Hall 301B 100 Foster Rd, Athens, GA 30602 USA

**Keywords:** Hypertension, Obesity, Abdominal obesity

## Abstract

**Objective:**

This study aimed to determine the association between body mass index (BMI) and abdominal obesity with hypertension among the South Asian adults (18–69 years).

**Methods:**

This study utilized the nationally representative WHO STEPwise approach to surveillance data (*n* = 24,413) from Afghanistan, Bangladesh, Bhutan, Nepal, and Sri Lanka. Hypertension was defined as having a systolic blood pressure of 140 mmHg or higher, a diastolic blood pressure of 90 mmHg or higher, and/or taking antihypertensive medications. A waist circumference ≥ 90 cm in men and ≥ 80 cm in women was considered as abdominal obesity. BMI was categorized according to Asia-specific cutoff and overweight was defined as BMI of 23.0–27.5 kg/m^2^ and obesity was defined as BMI ≥ 27.5 kg/m^2^. Multivariable logistic regression analyses were conducted to identify the association between BMI and abdominal obesity with hypertension. The odds ratio (OR) with a 95% confidence interval (CI) was reported.

**Results:**

Abdominal obesity increased the odds of hypertension 31%-105% compared to those who did not have abdominal obesity (OR: Afghanistan: 2.05; 95% CI: 1.27–3.31; Bangladesh: 1.55; 95% CI: 1.18–2.04; Bhutan: 1.31; 95% CI: 1.03–1.66; Nepal: 1.69; 95% CI: 1.31–2.18; Sri Lanka:1.55; 95% CI: 1.23–1.95). The odds increased among participants with both overweight/obesity and abdominal obesity. In all five countries under study, participants with both overweight and abdominal obesity (OR: Afghanistan: 2.75; 95% CI: 1.75–4.34; Bangladesh: 2.53; 95% CI: 1.90–3.37; Bhutan: 2.22; 95% CI: 1.64–3.00; Nepal: 2.08; 95% CI: 1.54–2.81; Sri Lanka: 2.29; 95% CI: 1.77–2.98), as well as those with obesity and abdominal obesity (OR: Afghanistan: 6.94; 95% CI: 4.68–10.30; Bangladesh: 2.95; 95% CI: 2.19–3.97; Bhutan: 3.02; 95% CI: 2.23–4.09; Nepal: 4.40; 95% CI: 3.05–6.34; Sri Lanka: 3.96; 95% CI: 2.94–5.32), exhibited higher odds of having hypertension as compared to participants with a normal BMI and no abdominal obesity.

**Conclusion:**

Having both abdominal obesity and overweight/obesity increased the odds of hypertension among South Asian adults. Preventing overweight/obesity and abdominal obesity is necessary for preventing the burden of hypertension in South Asia.

**Graphical Abstract:**

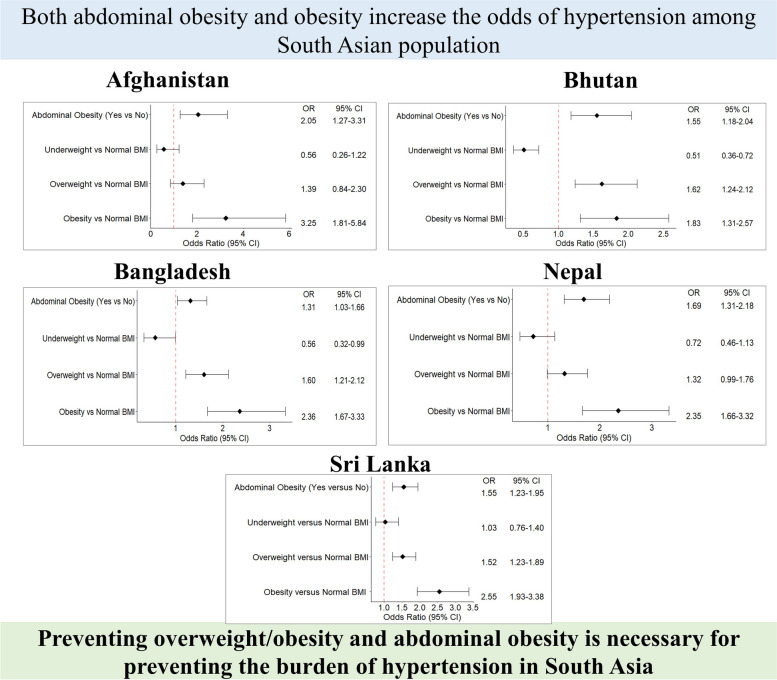

**Supplementary Information:**

The online version contains supplementary material available at 10.1186/s40885-023-00257-2.

## Introduction

Cardiovascular diseases (CVDs) are a leading cause of mortality worldwide [1]. The elevated body mass index (BMI), measured as the ratio of weight (in kilograms) to height (in meters) squared, is a surrogate marker for both total body fat and abdominal obesity [2–4]. Increased BMI and abdominal obesity are strongly associated with diabetes, CVD, and all-cause mortality [5, 6]. However, research suggests that abdominal obesity, stemming from the accumulation of visceral fat, is believed to underlie insulin resistance, which can trigger the development of atherosclerosis and hypertension—another significant risk factor for CVDs [7–9].

According to the Global Burden of Disease study, data from 195 countries reported that high systolic blood pressure accounted for 10.4 million deaths and 218 million disability-adjusted life years in 2017 [10]. Abdominal obesity and high BMI are also well-established risk factors for hypertension and CVDs.

The co-occurrence of these risk factors has become a significant public health concern, especially in South Asian countries, where the prevalence of obesity, abdominal obesity, and hypertension is on the rise [11–15]. The increasing prevalence of these risk factors is a cause for concern, as they are known to increase the risk of CVDs and mortality. The South Asian population is also known to be at a higher risk of CVDs, and this co-occurrence of risk factors is likely to exacerbate the risk [12–14]. Therefore, it is essential to understand the association between these risk factors and hypertension in this population and identify high-risk subgroups for targeted interventions.

The prevalence of abdominal obesity is estimated to be around 58% a men and 78% in women in the South Asian population [16]. The prevalence varies among the regions. Nationally representative studies found the prevalence ranging from 30.1% in Nepal to 57.71% in India [17, 18].

Despite the high burden of hypertension and obesity in the region, there is a significant gap in evidence regarding the combined effects of abdominal obesity and high BMI on hypertension among South Asian adults. Most of the available studies have focused on either hypertension or obesity, rather than exploring the combined effects of these two risk factors [19, 20]. Furthermore, most of the studies have been conducted in specific populations, such as urban areas or certain age groups, which may not be representative of the entire population. The limited evidence regarding the combined effects of abdominal obesity and high BMI on hypertension is a significant challenge for public health policy and intervention development. There is a need for studies that use nationally representative samples to examine the relationship between abdominal obesity, high BMI, and hypertension in South Asia.

The current study aims to address this gap by analyzing nationally representative data to identify the association between abdominal obesity, high BMI, and hypertension among South Asian adults. The objective of this study is to explore the association between the combined effect of abdominal obesity and high BMI and hypertension in South Asian adults using nationally representative data. We aim to investigate the prevalence of abdominal obesity and high BMI in South Asian adults and their association with hypertension. We also aim to explore the combined effect of these risk factors on hypertension and identify the population subgroups that are most affected by this co-occurrence of risk factors.

## Methods

### Study design, setting and sampling

A secondary analysis of the nationally representative WHO STEPwise approach to surveillance (STEPS) surveys was performed on data collected from five South Asian countries. We utilized data from the following surveys: Afghanistan 2018, Bangladesh 2017–18, Bhutan 2019, Nepal 2019, and Sri Lanka 2014. These surveys were implemented by the World Health Organization (WHO) to update the indicators related to non-communicable diseases in the low-and-middle-income countries. The detailed methods of each of these surveys including sample size calculation, sampling procedure, data collection and measurement, and findings were published previously [21–25].

### Data collection

The surveys employed multistage stratified cluster sampling to ensure representativeness. The first stage involved randomly selecting primary sampling units (PSUs) from both urban and rural areas using either probability proportional to size methods (in Afghanistan, Bhutan, Nepal, and Sri Lanka) or by selecting an equal number of PSUs from each division of the country (in Bangladesh). Following this, a predetermined number of households were then selected from each PSU, and participants were chosen from these households for data collection purposes. Trained enumerators collected the data in three stages: (i) questionnaire interview, (ii) physical measurement, and (iii) biochemical measurements [21–25]. Face-to-face interviews were conducted to collect the data related to sociodemographic and behavioral risk factors using a pre-tested and validated questionnaire. Next, calibrated instruments were used to conduct anthropometric measurements of the participant’s height, weight, waist circumference, and blood pressure. Blood and urine samples were collected during the final stage [21–25].

A portable stadiometer (Seca Measuring Scale 213®) was used to measure height. Participants were instructed to take off their shoes, slippers, or sandals and remove any headwear or hair bands. They stood on the stadiometer, facing the interviewer, feet close together and knees unbent. Their gaze was directed forward, ensuring their eyes aligned with their ears. The height was measured in centimeters. Using a Tanita digital scale positioned on a stable, even ground, weight was measured. Participants were advised to remove shoes and socks, don lightweight attire, and stand evenly distributed on the scale, looking straight with arms relaxed by their sides. They remained in position until directed to step down. Weights were measured in kilograms [21–25].

Using a uniform tension tape from Seca, Germany, waist circumference was measured. The measurements were taken in a secluded area of the residence and over lightweight clothing. The waist dimension was gauged halfway between the final noticeable rib and the apex of the iliac crest, with the individual’s arms casually by their side after exhaling normally. Participants were guided on the proper placement of the measuring tape. The values were recorded to an accuracy of 0.1 cm while maintaining the tape’s tension [21–25].

Blood pressure was measured using a digital BP–BOSO–Medicus Control monitor with a standard cuff size. Participants rested quietly for 15 min with legs uncrossed before measurements. After three readings, with a three-minute rest between each, the average of the last two readings was taken. The cuff was placed on the left arm, aligning with the brachial artery and ensuring its positioning 1.2 to 2.5 cm above the elbow’s inner side, with participants adjusting clothing as necessary [21–25].

The enumerators were recruited following standard procedures. All field staff attended comprehensive training sessions in their respective countries. The training session covered the survey protocol, questionnaire, field procedure manual, interview techniques, and physical measurements. It also addressed the aseptic collection, storage, and transportation of blood and urine samples, supervision of field activities, subject safety, privacy, confidentiality, and data collection. WHO technical experts facilitated the training. To maintain data quality, regular field supervision and daily data reviews were conducted. Medical technologists, trained to follow standard guidelines, collected biomedical samples. Participants were given detailed instructions on urine and fasting blood sample collection a day prior and provided with urine containers. Standard procedures were emphasized to participants for correct sample collection. Laboratory instruments were calibrated, and sample findings were cross-checked with another national standard laboratory [21–25].

### Outcome variable

The outcome of interest in this study was hypertension which was defined as having a systolic blood pressure (SBP) ≥ 140 mmHg and/or a diastolic blood pressure (DBP) ≥ 90 mmHg. If the participant was taking any antihypertensive medication at the time of the survey, the participant was considered to be hypertensive [26]. Hypertension was dichotomized into hypertensive/ normotensive.

### Independent variables

The main independent variables of interest were abdominal obesity (yes/no) and BMI (categorized into: normal weight, underweight, overweight, and obesity). A waist circumference ≥ 90 cm in men and ≥ 80 cm in women was considered as abdominal obesity [27]. BMI was defined as dividing the participant’s weight measured in kilograms by the square of the height measured in meters. An Asia-specific cut-off was used to categorize the BMI into the following categories: (a) BMI < 18.5 kg/m^2^: underweight; (b) 18.5 kg/m^2^ < BMI < 23.0 kg/m^2^: normal BMI; (c) 23.0 kg/m^2^ < BMI < 27.5 kg/m^2^: overweight; (d) BMI ≥ 27.5 kg/m^2^: obesity [28].

The covariates considered in this study were age, sex, highest educational attainment, current smoking status, current alcohol consumption, insufficient fruit and vegetable intake, inadequate physical activity, and diabetes. Age group was categorized into 18–29 years, 30–49 years, 50–69 years [17, 29, 30], and the highest educational attainment was categorized into no formal schooling, up to primary, up to secondary, and college and higher. Current smoking status was categorized into three categories: never smoker, current smoker, and former smoker. Current alcohol consumption was defined as drinking alcohol within 30 days of the survey [21–25]. It was dichotomized into yes and no. Insufficient fruit and vegetable intake was defined as consuming less than 5 servings of fruits and vegetables per day [21–25]. It was dichotomized into yes and no. Adequate physical activity was defined as engaging in a minimum of 150 min per week of moderate-intensity aerobic physical activity or a minimum of 75 min per week of vigorous-intensity aerobic physical activity [31]. Diabetes was defined as a fasting blood glucose level ≥ 126 mg/dl and/or taking medications for diabetes [21–25].

### Statistical analysis

At first descriptive analyses were conducted. For continuous variables, the findings were reported in mean with standard deviation. For categorical variables, the findings were reported in unweighted frequency and weighted percentages. To find the association between hypertension with abdominal obesity and BMI, multivariable logistic regression analyses were conducted. We conducted three models. Model 1 adjusted for abdominal obesity and covariates, Model 2 adjusted for BMI and covariates, and Model 3 adjusted for both abdominal obesity and BMI. Also, we conducted a separate analysis to see the joint effect of abdominal obesity and BMI on hypertension. We categorized the participants into eight groups: (i) no abdominal obesity, Normal BMI; (ii) abdominal obesity, Normal BMI; (iii) no abdominal obesity, underweight; (iv) abdominal obesity, underweight; (v) no abdominal obesity, overweight; (vi) abdominal obesity, overweight; (vii) no abdominal obesity, obesity; (viii) abdominal obesity, obesity. The category of ‘no abdominal obesity, Normal BMI’ was used as the reference group. The odds of hypertension in other groups were estimated. Given the absence of a standardized Asia-specific cut-off for waist circumference, we conducted a sensitivity analysis using a threshold of ≥ 78 cm for men and ≥ 72 cm for women, as proposed by Misra et al. for the Asian Indian population [32].

The findings of the logistic regression analyses were reported in odds ratio (OR) with 95% confidence interval (CI). During the analyses, the sample weights of WHO STEPS were adjusted. Stata Version 17.0 and R version 4.2.3. were used for the analyses.

### Ethical considerations

The country specific institutional review boards of the respective countries approved the STEPS protocols (Afghanistan: Ministry of Public Health Ethics Board; Bangladesh: Bangladesh Medical Research Council; Bhutan: Research Ethics Board for Health; Nepal: Nepal Health Research Council; Sri Lanka: Medical Research Institute) [21–25]. Prior to data collection, written informed consent was taken from the participants.

## Results

The background characteristics of the participants and prevalence of hypertension according to covariates are shown in Table 1. In total, 24,413 participants were included in the study from five countries. The range of the prevalence of overweight, obesity, and abdominal obesity were 28.77%-39.21%, 12.16%-26.43%, and 27.64%-53.55%, respectively. The prevalence of hypertension ranged from 21.56% to 30.74% (Afghanistan: 30.74%; Bangladesh: 21.56%; Bhutan: 28.64%; Nepal: 26.79%; Sri Lanka: 27.47%). In all five counties, the prevalence of hypertension increased with increasing age (*p* < 0.001) and BMI (*p* < 0.001). The prevalence was significantly higher among those having diabetes mellitus and abdominal obesity compared to those who did not have diabetes (*p* < 0.001) and abdominal obesity (*p* < 0.001). The detailed background characteristics are shown in Supplementary Tables 1, 2, 3, 4, and 5.

**Table 1:** Background characteristics of the participants and prevalence of hypertension according to covariates

Variables	Afghanistan (*n* = 3,350)		Bangladesh (*n* = 6,847)		Bhutan (*N* = 5,164)		Nepal (*n* = 4,894)		Sri Lanka (*n* = 4,158)	
	**Hypertensive**	**Normotensive**	**Hypertensive**	**Normotensive**	**Hypertensive**	**Normotensive**	**Hypertensive**	**Normotensive**	**Hypertensive**	**Normotensive**
**Age group**										
18–29	22.02	77.98	9.7	90.3	10.81	89.19	14.9	85.1	9.88	90.12
30–49	32.22	67.78	23.34	76.66	33.99	66.01	28.14	71.86	24.54	75.46
50–69	50.2	49.8	38.49	61.51	51.57	48.43	45.74	54.26	51.69	48.31
*p*-value	< 0.001		< 0.001		< 0.001		< 0.001		< 0.001	
**Gender**										
Male	26.24	73.76	17.33	82.67	32.59	67.41	32.37	67.63	25.82	74.18
Female	36.84	63.16	25.79	74.21	23.91	76.09	21.83	78.17	28.55	71.45
*p*-value	< 0.01		< 0.001		< 0.001		< 0.001		0.1074	
**Education**										
No formal schooling	36.21	63.79	24.53	75.47	35.76	64.24	33.02	66.98	39.83	60.17
Up to primary	32.83	67.17	20.09	79.91	31.06	68.94	29.05	70.95	34.11	65.89
Up to secondary	17.75	82.25	20.48	79.52	18.21	81.79	18.96	81.04	25.9	74.1
College and higher	9.34	90.66	20.4	79.6	32.5	67.5	24.63	75.37	21.54	78.46
*p*-value	0.055		0.055		0.055		0.055		0.055	
**Current smoking**										
Yes	26.95	73.05	14.16	85.84	22.01	77.99	32.72	67.28	25.75	74.25
No	31.25	68.75	23.94	76.06	29.42	70.58	25.48	74.52	27.37	72.63
*p*-value	0.5514		< 0.001		0.0124		0.003		0.5198	
**Alcohol use in last 30 days**										
Yes	50.07	49.93	13.42	86.58	33.21	66.79	36.7	63.3	29.48	70.52
No	30.74	69.26	22.35	77.65	21.65	78.35	22.65	77.35	26	74
*p*-value	0.232		0.0024		< 0.001		< 0.001		0.0598	
**Sufficient fruit and vegetable intake**										
Yes	29.37	70.63	18.99	81.01	27.29	72.71	22.96	77.04	29.28	70.72
No	31.04	68.96	22.34	77.66	29.44	70.56	27.33	72.67	25.1	74.9
*p*-value	0.7622		0.0506		0.2175		0.2034		0.0194	
**Adequate physical activity**										
Yes	24.43	75.57	14.29	85.71	24.51	75.49	26.72	73.28	21.66	78.34
No	31.82	68.18	23.2	76.8	29.7	70.3	26.8	73.2	27.74	72.26
*p*-value	0.1282		< 0.001		0.0223		0.9788		0.0472	
**Diabetes**										
No	26.73	73.27	19.18	80.82	27.79	72.21	25.6	74.4	24.18	75.82
Yes	62.55	37.45	49.38	50.62	59.15	40.85	45.25	54.75	53.05	46.95
*p*-value	< 0.001		< 0.001		< 0.001		< 0.001		< 0.001	
**BMI category**										
Under weight	10.39	82.5	9.16	84.28	8.41	81.91	16.5	78.32	17.21	80.5
Normal Weight	17.5	89.61	15.72	90.84	18.09	91.59	21.68	83.5	19.5	82.79
Overweight	29.06	70.94	29.17	70.83	29.95	70.05	29.82	70.18	32.55	67.45
Obesity	55.26	44.74	38.96	61.04	43.36	56.64	46.83	53.17	47.68	52.32
*p*-value	< 0.001		< 0.001		< 0.001		< 0.001		< 0.001	
**Abdominal Obesity**										
No	16.08	83.92	15.19	84.81	22.96	77.04	22.12	77.88	19.96	80.04
Yes	43.66	56.34	38.22	61.78	38.21	61.79	37.61	62.39	38.3	61.7
*p*-value	< 0.001		< 0.001		< 0.001		< 0.001		< 0.001	
**Abdominal obesity, body mass index**										
No, Normal	13.95	86.05	14.27	85.73	17.82	82.18	20.81	79.19	18.59	81.41
No, Underweight	7.25	92.75	9.14	90.86	8.11	91.89	16.45	83.55	16.85	83.15
No, Overweight	20.27	79.73	22.48	77.52	27.95	72.05	27.3	72.7	26.15	73.85
No, Obesity	33.94	66.06	28.6	71.4	45.89	54.11	29.32	70.68	25.76	74.24
Yes, Normal	28.2	71.8	40.76	59.24	22.27	77.73	27.12	72.88	23.32	76.68
Yes, Underweight	22.37	77.63	10.08	89.92	25.24	74.76	18.56	81.44	23.16	76.84
Yes, Overweight	34.99	65.01	36.96	63.04	33.5	66.5	33.06	66.94	37.01	62.99
Yes, Obesity	57.67	42.33	39.83	60.17	42.91	57.09	51.22	48.78	50.63	49.37
*p*-value	< 0.001		< 0.001		< 0.001		< 0.001		< 0.001	

Table 2 and Fig. 1.1–1.5 show the logistics regression result regarding the association between BMI, abdominal obesity, and hypertension. The full models are shown in Supplementary Tables 6, 7, 8, 9, and 10. In all five countries, obesity was significantly associated with increased odds of hypertension. In Bangladesh, Bhutan, and Sri Lanka, overweight was significantly associated with increased odds of hypertension. Compared to normal BMI, obesity increased the odds of hypertension 83%-225% (OR: Afghanistan: 3.25; 95% CI: 1.81–5.84; Bangladesh: 1.83; 95% CI: 1.31–2.57; Bhutan: 2.36; 95% CI: 1.67–3.33; Nepal: 2.35; 95% CI: 1.66–3.32; Sri Lanka: 2.55; 95% CI: 1.93–3.38). In all five countries, even after adjusting for BMI, abdominal obesity was significantly associated with hypertension. Abdominal obesity increased the odds of hypertension 31%-105% compared to those who did not have abdominal obesity (OR: Afghanistan: 2.05; 95% CI: 1.27–3.31; Bangladesh: 1.55; 95% CI: 1.18–2.04; Bhutan: 1.31; 95% CI: 1.03–1.66; Nepal: 1.69; 95% CI: 1.31–2.18; Sri Lanka:1.55; 95% CI: 1.23–1.95).

**Table 2:** Logistics regression finding regarding the association between BMI, abdominal obesity and hypertension

Variables	Afghanistan		Bangladesh		Bhutan		Nepal		Sri Lanka	
	Model 1	Model 2	Model 1	Model 2	Model 1	Model 2	Model 1	Model 2	Model 1	Model 2
Abdominal Obesity										
No	Ref	Ref	Ref	Ref	Ref	Ref	Ref	Ref	Ref	Ref
Yes	3.36^***^ (2.50–4.51)		2.43^***^ (1.96–3.02)		2.02^***^ (1.66–2.46)		2.43^***^ (1.95–3.02)		2.24^***^ (1.84–2.72)	
BMI category										
Normal Weight	Ref	Ref	Ref	Ref	Ref	Ref	Ref	Ref	Ref	Ref
Under weight		0.53 (0.23–1.19)		0.50^***^ (0.36–0.70)		0.55^*^ (0.31–0.96)		0.67 (0.43–1.05)		0.97 (0.72–1.31)
Overweight		1.76* (1.23–2.70)		1.95^***^ (1.55–2.46)		1.72^***^ (1.32–2.25)		1.53^***^ (1.17–1.99)		1.80^***^ (1.47–2.20)
Obesity		4.75^***^ (5.23–7.30)		2.61^***^ (1.99–3.43)		2.86^***^ (2.14–3.82)		3.24^***^ (2.37–4.43)		3.31^***^ (2.56–4.29)

Model 1: Adjusted for abdominal obesity and covariates (age group, gender, education, current smoking, alcohol use in last 30 days, sufficient fruit and vegetable intake, adequate physical activity, diabetes)

Model 2: Adjusted for BMI category and covariates (age group, gender, education, current smoking, alcohol use in last 30 days, sufficient fruit and vegetable intake, adequate physical activity, diabetes)

^*^p < 0.05

^***^p < 0.001

**Fig. 1:**
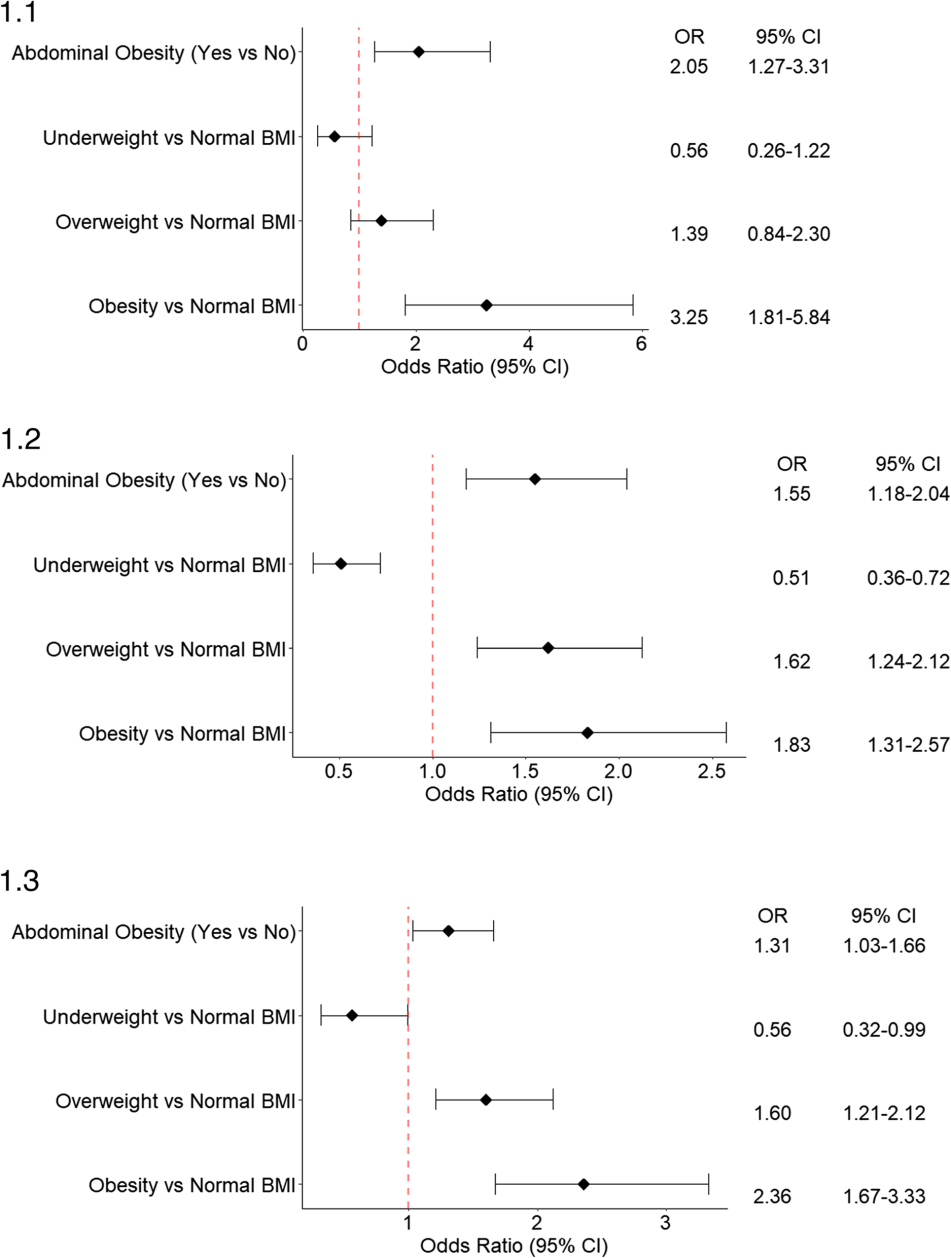

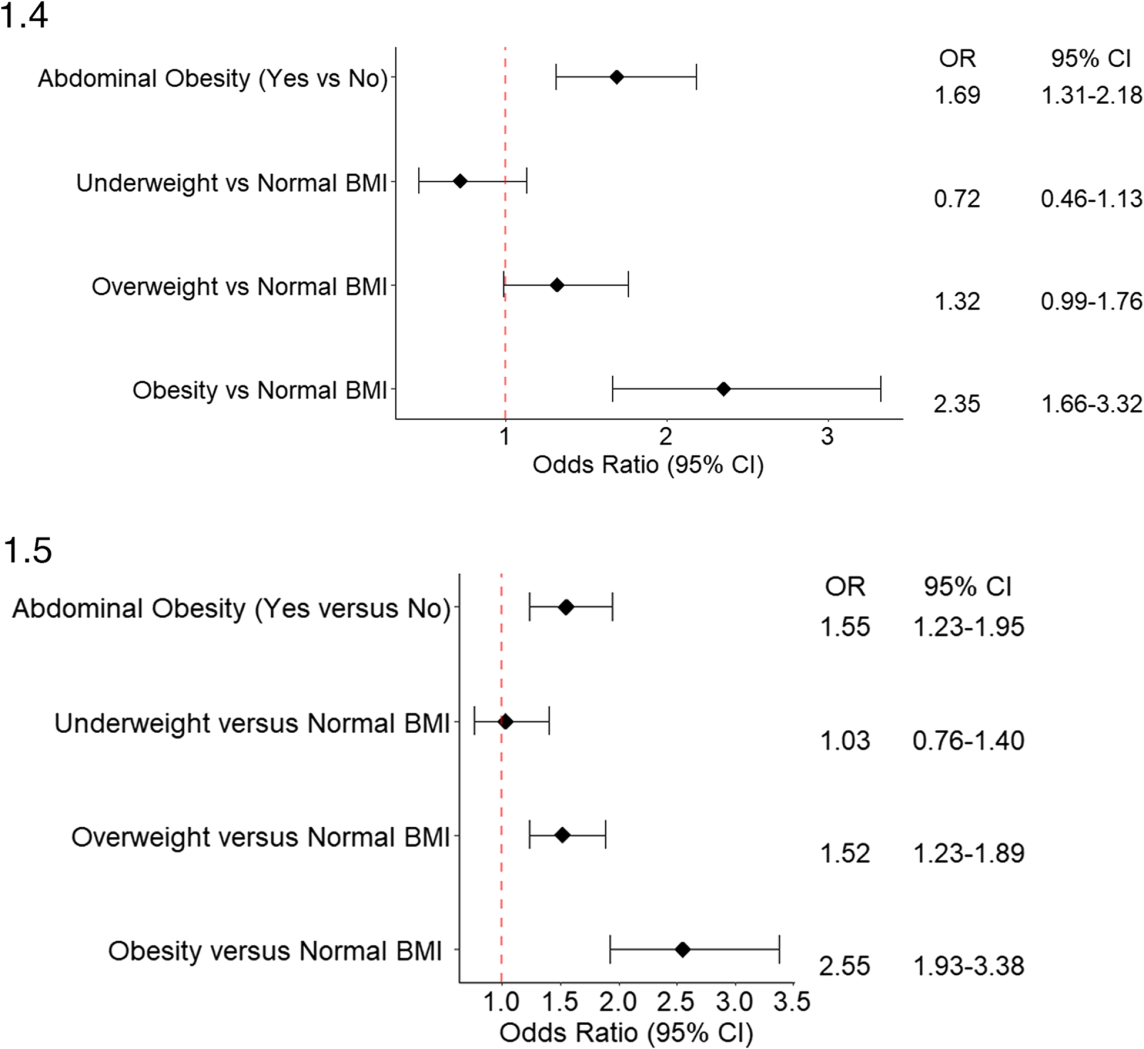
Analysis of the Relationship between BMI, Abdominal Obesity, and Hypertension (Adjusted for both BMI and abdominal obesity, age group, gender, education, current smoking, alcohol use in last 30 days, sufficient fruit and vegetable intake, adequate physical activity, diabetes). 1.1: Analysis of the Relationship between BMI, Abdominal Obesity, and Hypertension in Afghanistan. 1.2: Analysis of the Relationship between BMI, Abdominal Obesity, and Hypertension in Bangladesh. 1.3: Analysis of the Relationship between BMI, Abdominal Obesity, and Hypertension in Bhutan. 1.4: Analysis of the Relationship between BMI, Abdominal Obesity, and Hypertension in Nepal. 1.5: Analysis of the Relationship between BMI, Abdominal Obesity, and Hypertension in Sri Lanka

The logistics regression findings regarding the association between the combined effect of BMI and abdominal obesity with hypertension are shown in Fig. 2.1–2.5. In all five countries under study, participants with both overweight and abdominal obesity (OR: Afghanistan: 2.75; 95% CI: 1.75–4.34; Bangladesh: 2.53; 95% CI: 1.90–3.37; Bhutan: 2.22; 95% CI: 1.64–3.00; Nepal: 2.08; 95% CI: 1.54–2.81; Sri Lanka: 2.29; 95% CI: 1.77–2.98), as well as those with obesity and abdominal obesity (OR: Afghanistan: 6.94; 95% CI: 4.68–10.30; Bangladesh: 2.95; 95% CI: 2.19–3.97; Bhutan: 3.02; 95% CI: 2.23–4.09; Nepal: 4.40; 95% CI: 3.05–6.34; Sri Lanka: 3.96; 95% CI: 2.94–5.32), exhibited higher odds of having hypertension as compared to participants with a normal BMI and no abdominal obesity. The odds of hypertension were higher among the participants with a normal BMI with abdominal obesity, significant association was observed among the participants in Bangladesh (OR: 3.18; 95% CI: 1.83–5.54) and Nepal (OR: 1.54; 95% CI: 1.03–2.28). Similar findings were revealed in the sensitivity analyses (Supplementary Tables 11, 12, 13, 14 and 15).

**Fig. 2:**
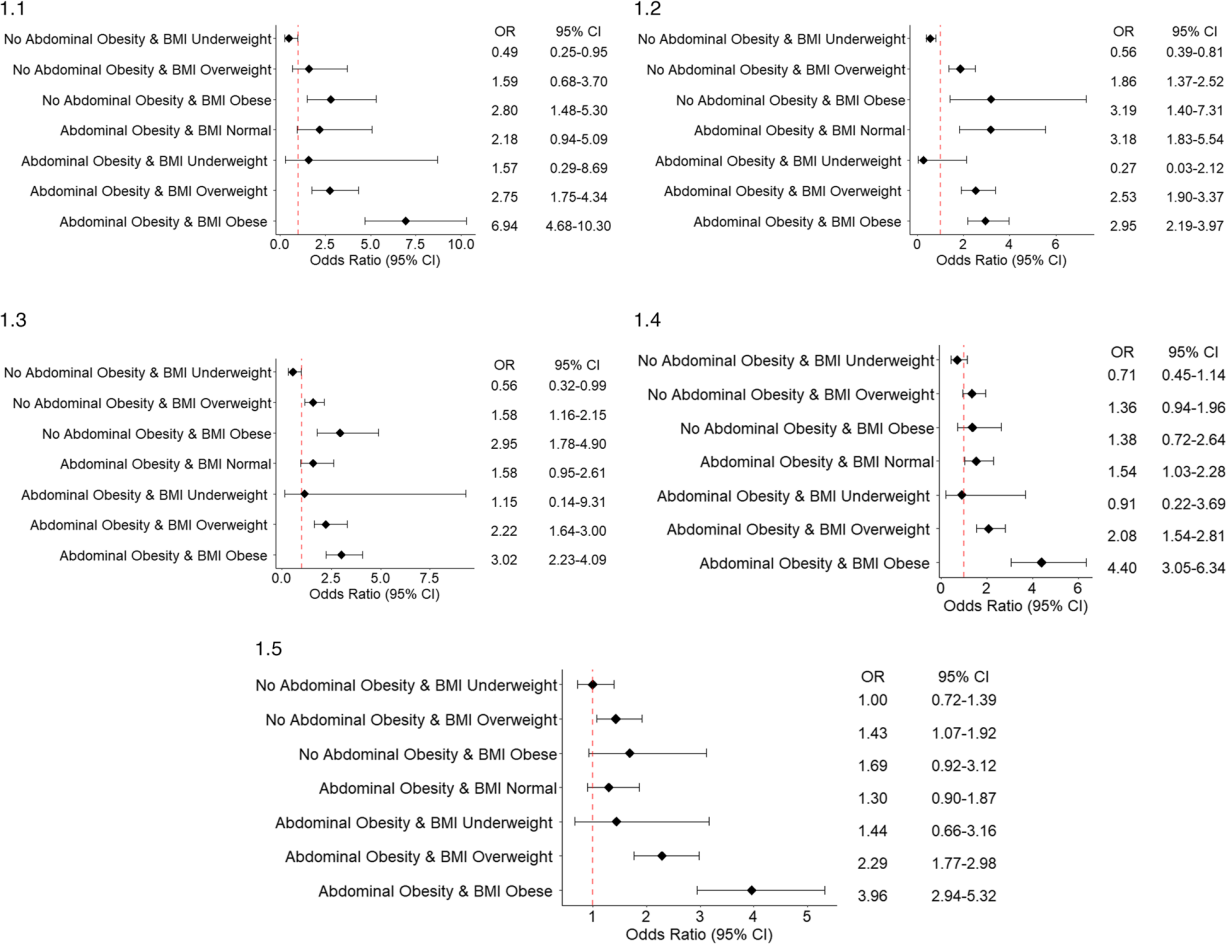
The association between the combined effect of BMI and abdominal obesity with hypertension (Adjusted for age group, gender, education, current smoking, alcohol use in last 30 days, sufficient fruit and vegetable intake, adequate physical activity, diabetes). 2.1: The association between the combined effect of BMI and abdominal obesity with hypertension in Afghanistan. 2.2: The association between the combined effect of BMI and abdominal obesity with hypertension in Bangladesh. 2.3: The association between the combined effect of BMI and abdominal obesity with hypertension in Bhutan. 2.4: The association between the combined effect of BMI and abdominal obesity with hypertension in Nepal. 2.5: The association between the combined effect of BMI and abdominal obesity with hypertension in Sri Lanka

## Discussion

This study aims to determine the association between BMI and abdominal obesity with hypertension in five South Asian countries. Among the study participants, the findings from the nationally representative data showed that after adjusting BMI, abdominal obesity is independently associated with hypertension. The combined effects of high BMI and abdominal obesity generates higher odds of having hypertension compared to normal BMI and no abdominal obesity across the population from Afghanistan, Bangladesh, Bhutan, Nepal and Sri Lanka. Despite being of normal BMI, people with abdominal obesity from Bangladesh and Nepal presented with significant increased odds of hypertension.

These findings are consistent with systematic reviews published by Zhou et al. [30] and Jayedi et al. [31]. According to their findings, every 5 units and 10 cm increase in BMI and WC can increase the odds of hypertension by 49–50% and 25–27%, respectively [2, 33]. Besides, in accordance with our study, the combined effects of high BMI and abdominal obesity has high specificity in predicting hypertension as shown from a study by Peixoto et al. in 2006 [34]. The findings are also consistent with upper income countries such as the United States, where individuals with normal BMI with abdominal obesity yielded higher risk of being hypertensive compared to their counterparts without abdominal obesity [35]. Similar increased risk of hypertension among the people with normal BMI with abdominal obesity was noticed among the Chinese population [36].

Previous studies found that WC exhibited greater ability to predict cardiometabolic risk compared to BMI [37]. The adipose tissue dysfunction is evidenced by hypertrophied adipocytes, enhanced macrophage infiltration, and significant alterations in adipokine and free fatty acid release causing persistent vascular inflammation, oxidative stress, renin–angiotensin–aldosterone system activation, and sympathetic overdrive, finally leading to hypertension [38, 39]. This condition became particularly worse for Asian population due to higher proportion of body fat and predominant abdominal obesity compared to European people as described by Yudkin-Yajnik (The Y-Y) paradox [40, 41].

There are several mechanisms through which obesity and abdominal obesity lead to hypertension. These include complex interactions among the renal, metabolic, and neuroendocrine systems. Obesity results in the overactivation of the Sympathetic Nervous System (SNS) due to adipokine secretion, stimulation of the Renin–Angiotensin–Aldosterone System (RAAS), insulin resistance, baroreceptor dysfunction, and obstructive sleep apnea. Overactivation of the SNS leads to an elevated heart rate, increased cardiac output, and enhanced renal sodium reabsorption [42, 43].

Obesity and abdominal obesity also cause activation of the RAAS due to the SNS-RAAS interaction, physical compression of the kidney by visceral fat, and the direct release of angiotensinogen by adipocytes. This activation of RAAS triggers systemic vasoconstriction and water retention in the body, which ultimately leads to a rise in blood pressure [44, 45].

Furthermore, obesity often results in renal compression and inflammation [46]. As a compensatory mechanism, there is an increase in both blood pressure and renal filtration. However, these changes might culminate in kidney damage and, subsequently, hypertension. Intensifying this challenge, the renal sodium reabsorption process receives an added boost both directly and indirectly through the SNS and RAAS [47–49].

In cases of obesity and abdominal obesity, leptin levels increase in the blood. High leptin levels, rather than suppressing appetite, might stimulate the SNS and thus play a role in elevating blood pressure [50]. Lastly, obesity and abdominal obesity are associated with insulin resistance, which leads to overstimulation of the SNS and sodium retention, resulting in hypertension [51, 52].

According to the WHO, low- and middle-income nations are disproportionately affected by hypertension, which affects not only health but all aspects of social and economic development [53]. In the WHO South-East Asia Region, 25% of adults have high blood pressure, yet only one in three are receiving treatment, and only one in ten of these persons have their condition under control [53]. In 2015, approximately 9.5 million deaths were recorded due to hypertension and 88% of them were from low-income and middle-income countries resulting from doubling of high blood pressure deaths from South Asia, South-East Asia and Sub-Saharan Africa within 15 years since 1990 [52].

WHO is operating prevention and control of hypertension as part of their non-communicable disease program by actively participating in India, Bangladesh, Nepal, and Sri Lanka in co-operation with local governments. Although South Asia along with Sub-Saharan Africa and Oceania shares the lowest rates of detection, treatment, and control of hypertension, researchers reported that reduction of hypertension prevalence through preventive and control measures is possible in low-income and middle-income countries [53]. This low level of diagnosis and therapeutic capability provides an opportunity for stakeholders to improve health coverage by emphasizing the population at risk, as defined by presence of abdominal obesity and high BMI.

This study utilized nationally representative samples, enabling its findings to be generalizable to the target population. There are several limitations of this study. Due to the cross-sectional nature of the WHO-STEPS survey, we could not establish temporal relationship between the exposure and the outcome. The information related to wealth index/income was not present at the datasets, so we could not adjust wealth index/income in our analysis.

## Conclusion

The study found that abdominal obesity is associated with increased odds of hypertension among South Asian population. The combined effect of overweight/obesity and abdominal obesity exhibited higher odds of hypertension compared to the participants ‘with normal BMI and without abdominal obesity’. The odds of hypertension were also higher among the participants with normal BMI and abdominal obesity. Health promotion programs in South Asian should emphasize preventing both overweight/obesity and abdominal obesity in order to prevent the burden of hypertension.

### Supplementary Information

**Additional file 1: Table 1.** Distribution of the background characteristics of the participants in Afghanistan (*n*= 3,351). **Table 2.** Distribution of the background characteristics of the participants in Bangladesh (*n*=6,847). **Table 3.** Distribution of the background characteristics of the participants in Bhutan (*n*=5,164). **Table 4.** Distribution of the background characteristics of the participants in Nepal (*n*=4,894). **Table 5.** Distribution of the background characteristics of the participants in Sri Lanka (*n*=4,158). **Table 6.** Findings from the logistic regression models showing factors associated with hypertension in Afghanistan. **Table 7.** Findings from the logistic regression models showing factors associated with hypertension in Bangladesh. **Table 8.** Findings from the logistic regression models showing factors associated with hypertension in Bhutan. **Table 9.** Findings from the logistic regression models showing factors associated with hypertension in Nepal. **Table 10.** Findings from the logistic regression models showing factors associated with hypertension in Sri Lanka. **Table 11.** Findings from the logistic regression models showing factors associated with hypertension in Afghanistan. **Table 12.** Findings from the logistic regression models showing factors associated with hypertension in Bangladesh. **Table 13.** Findings from the logistic regression models showing factors associated with hypertension in Bhutan. **Table 14.** Findings from the logistic regression models showing factors associated with hypertension in Nepal. **Table 15.** Findings from the logistic regression models showing factors associated with hypertension in Sri Lanka.

## Data Availability

The data of WHO STEPS survey is available at NCD Microdata Repository (url: https://extranet.who.int/ncdsmicrodata/index.php/home). The data can be accessed following proper procedure.

## Abbreviations

BMI: Body Mass Index
CI: Confidence Interval
OR: Odds Ratio
WHO: World Health Organization
