# Supplementary material for: The association between body mass index and abdominal obesity with hypertension among South Asian population: findings from nationally representative surveys
Source: Clin Hypertens. 2024 Feb 1;30:3. doi: 10.1186/s40885-023-00257-2 (PMC10832172; doi:10.1186/s40885-023-00257-2)
Supplement: Supplementary file 1 — Additional file 1: Table 1. Distribution of the background characteristics of the participants in Afghanistan (n= 3,351). Table 2. Distribution of the background characteristics of the participants in Bangladesh (n=6,847). Table 3. Distribution of the background characteristics of the participants in Bhutan (n=5,164). Table 4. Distribution of the background characteristics of the participants in Nepal (n=4,894). Table 5. Distribution of the background characteristics of the participants in Sri Lanka (n=4,158). Table 6. Findings from the logistic regression models showing factors associated with hypertension in Afghanistan. Table 7. Findings from the logistic regression models showing factors associated with hypertension in Bangladesh. Table 8. Findings from the logistic regression models showing factors associated with hypertension in Bhutan. Table 9. Findings from the logistic regression models showing factors associated with hypertension in Nepal. Table 10. Findings from the logistic regression models showing factors associated with hypertension in Sri Lanka. Table 11. Findings from the logistic regression models showing factors associated with hypertension in Afghanistan. Table 12. Findings from the logistic regression models showing factors associated with hypertension in Bangladesh. Table 13. Findings from the logistic regression models showing factors associated with hypertension in Bhutan. Table 14. Findings from the logistic regression models showing factors associated with hypertension in Nepal. Table 15. Findings from the logistic regression models showing factors associated with hypertension in Sri Lanka. [file 40885_2023_257_MOESM1_ESM.docx]

**Table 1: Distribution of the background characteristics of the participants in Afghanistan (n= 3,351)**

| **Variables** | **Total** | **Normal BP** | **Hypertension** | **p-value** |
| --- | --- | --- | --- | --- |
|  | **Frequency (Percentage)** | **Weighted Percentage** | **Weighted Percentage** |  |
| Weight in kg, mean (SD) | 67.12 (31.82) | 64.80 (36.41) | 72.34 (15.80) |  |
| Height in m, mean (SD) | 1.63 (0.27) | 1.64 (0.31) | 1.62 (0.01) |  |
| BMI in kg/m2, mean (SD) | 25.11 (6.05) | 23.95 (5.72) | 27.71 (5.96) |  |
| Systolic blood pressure in mmHg, mean (SD) | 126.31 (19.92) | 119.20 (10.65) | 142.25 (26.00) |  |
| Diastolic blood pressure in mmHg, mean (SD) | 80.81 (15.81) | 75.97 (7.94) | 91.66 (22.52) |  |
| **Age group** |  |  |  | <0.001 |
| 18-29 | 1195 (43.66) | 77.98 | 22.02 |  |
| 30-49 | 1349 (39.19) | 67.78 | 32.22 |  |
| 50-69 | 807 (17.15) | 49.8 | 50.2 |  |
| **Gender** |  |  |  | <0.01 |
| Male | 1834 (56.49) | 73.76 | 26.24 |  |
| Female | 1517 (43.51) | 63.16 | 36.84 |  |
| **Education** |  |  |  | 0.055 |
| No formal schooling | 1862 (58.79) | 63.79 | 36.21 |  |
| Up to primary | 585 (16.84) | 67.17 | 32.83 |  |
| Up to secondary | 743 (20.91) | 82.25 | 17.75 |  |
| College and higher | 161 (3.46) | 90.66 | 9.34 |  |
| **Current smoking** |  |  |  | 0.5514 |
| Yes | 308 (9.30) | 73.05 | 26.95 |  |
| No | 3043 (90.70) | 68.75 | 31.25 |  |
| **Alcohol use in last 30 days** |  |  |  | 0.232 |
| Yes | 42 (0.56) | 49.93 | 50.07 |  |
| No | 3309 (99.44) | 69.26 | 30.74 |  |
| **Sufficient fruit and vegetable intake** |  |  |  | 0.7622 |
| Yes | 410 (11.51) | 70.63 | 29.37 |  |
| No | 2941 (88.49) | 68.96 | 31.04 |  |
| **Adequate physical activity** |  |  |  | 0.1282 |
| Yes | 441 (13.14) | 75.57 | 24.43 |  |
| No | 2910 (86.86) | 68.18 | 31.82 |  |
| **Diabetes** |  |  |  | <0.001 |
| No | 2964 (88.48) | 73.27 | 26.73 |  |
| Yes | 387 (11.52) | 37.45 | 62.55 |  |
| **BMI category** |  |  |  | <0.001 |
| Normal Weight | 1027 (31.88) | 82.5 | 17.5 |  |
| Under weight | 232 (7.76) | 89.61 | 10.39 |  |
| Overweight | 1122 (33.93) | 70.94 | 29.06 |  |
| Obesity | 970 (26.43) | 44.74 | 55.26 |  |
| **Abdominal Obesity** |  |  |  | <0.001 |
| No | 1558 (46.45) | 83.92 | 16.08 |  |
| Yes | 1793 (53.55) | 56.34 | 43.66 |  |
| **Abdominal obesity, body mass index** |  |  |  | <0.001 |
| No, Normal | 821 (23.94) | 86.05 | 13.95 |  |
| No, Underweight | 194 (6.15) | 92.75 | 7.25 |  |
| No , Overweight | 442 (13.67) | 79.73 | 20.27 |  |
| No, Obesity | 101 (2.68) | 66.06 | 33.94 |  |
| Yes, Normal | 206 (7.94) | 71.8 | 28.2 |  |
| Yes, Underweight | 38 (1.61) | 77.63 | 22.37 |  |
| Yes, Overweight | 680 (20.26) | 65.01 | 34.99 |  |
| Yes, Obesity | 869 (23.75) | 42.33 | 57.67 |  |

**Table 2: Distribution of the background characteristics of the participants in Bangladesh (n=6,847)**

| **Variables** | **Total (n= 6,847 )** | **Normal BP** | **Hypertension** | p-value |
| --- | --- | --- | --- | --- |
|  | **Unweighted Frequency** | **Weighted Percentage** | **Weighted Percentage** |  |
| Weight in kg, mean (SD) | 55.58 | 54.70 (10.96) | 58.78 (12.76) |  |
| Height in m, mean (SD) | 1.57 | 1.58 (0.09) | 1.55 (0.10) |  |
| BMI in kg/m2, mean (SD) | 22.57 | 22.03 (4.07) | 24.51 (4.82) |  |
| Systolic blood pressure in mmHg, mean (SD) | 120.41 | 113.84 (10.02) | 144.31 (21.22) |  |
| Diastolic blood pressure in mmHg, mean (SD) | 78.40 | 74.33 (8.20) | 93.23 (11.33) |  |
| **Age group** |  |  |  | <0.001 |
| 18-29 | 1464 (39.19) | 90.3 | 9.7 |  |
| 30-49 | 3816 (37.28) | 76.66 | 23.34 |  |
| 50-69 | 1567 (23.52) | 61.51 | 38.49 |  |
| **Gender** |  |  |  | <0.001 |
| Male | 3217 (49.97) | 82.67 | 17.33 |  |
| Female | 3630 (50.03) | 74.21 | 25.79 |  |
| **Education** |  |  |  | 0.055 |
| No formal schooling | 2159 (31.13) | 75.47 | 24.53 |  |
| Up to primary | 3165 (45.99) | 79.91 | 20.09 |  |
| Up to secondary | 1103 (17.89) | 79.52 | 20.48 |  |
| College and higher | 420 (4.99) | 79.6 | 20.4 |  |
| **Current smoking** |  |  |  | <0.001 |
| Yes | 1647 (24.40) | 85.84 | 14.16 |  |
| No | 5200 (75.60) | 76.06 | 23.94 |  |
| **Alcohol use in last 30 days** |  |  |  | 0.0024 |
| Yes | 517 (8.87) | 86.58 | 13.42 |  |
| No | 6330 (91.13) | 77.65 | 22.35 |  |
| **Sufficient fruit and vegetable intake** |  |  |  | 0.0506 |
| Yes | 1624 (23.30) | 81.01 | 18.99 |  |
| No | 5223 (76.70) | 77.66 | 22.34 |  |
| **Adequate physical activity** |  |  |  | <0.001 |
| Yes | 1108 (18.41) | 85.71 | 14.29 |  |
| No | 5739 (81.59) | 76.8 | 23.2 |  |
| **Diabetes** |  |  |  | <0.001 |
| No | 6196 (92.12) | 80.82 | 19.18 |  |
| Yes | 651 (7.88) | 50.62 | 49.38 |  |
| **BMI category** |  |  |  | <0.001 |
| Normal Weight | 2757 (43.88) | 84.28 | 15.72 |  |
| Under weight | 917 (14.72) | 90.84 | 9.16 |  |
| Overweight | 2152 (28.77) | 70.83 | 29.17 |  |
| Obesity | 1021 (12.63) | 61.04 | 38.96 |  |
| **Abdominal Obesity** |  |  |  | <0.001 |
| No | 4581 (72.36) | 84.81 | 15.19 |  |
| Yes | 2266 (27.64) | 61.78 | 38.22 |  |
| **Abdominal obesity, body mass index** |  |  |  | <0.001 |
| No, Normal | 2558 (41.47) | 85.73 | 14.27 |  |
| No, Underweight | 904 (14.43) | 90.86 | 9.14 |  |
| No , Overweight | 1051 (15.48) | 77.52 | 22.48 |  |
| No, Obesity | 68 (0.98) | 71.4 | 28.6 |  |
| Yes, Normal | 199 (2.41) | 59.24 | 40.76 |  |
| Yes, Underweight | 13 (0.29) | 89.92 | 10.08 |  |
| Yes, Overweight | 1101 (13.29) | 63.04 | 36.96 |  |
| Yes, Obesity | 953 (11.65) | 60.17 | 39.83 |  |

**Table 3: Distribution of the background characteristics of the participants in Bhutan (n=5,164)**

| **Variables** | **Total (n= 5,164 )** | **Normal BP** | **Hypertension** | p-value |
| --- | --- | --- | --- | --- |
|  | **Unweighted Frequency** | **Weighted Percentage** | **Weighted Percentage** |  |
| Weight in kg, mean (SD) | 62.20 (11.73) | 60.63 (10.92) | 66.10 (12.83) |  |
| Height in m, mean (SD) | 1.58 (0.08) | 1.58 (0.08) | 1.58 (0.09) |  |
| BMI in kg/m2, mean (SD) | 24.97 (4.34) | 24.35 (4.08) | 26.53 (4.56) |  |
| Systolic blood pressure in mmHg, mean (SD) | 124.59 (16.15) | 117.65 (9.83) | 141.86 (16.44) |  |
| Diastolic blood pressure in mmHg, mean (SD) | 82.28 (11.46) | 77.14 (7.21) | 95.10 (10.21) |  |
| **Age group** |  |  |  | <0.001 |
| 18-29 | 1170 (37.04) | 89.19 | 10.81 |  |
| 30-49 | 2654 (44.52) | 66.01 | 33.99 |  |
| 50-69 | 1340 (18.44) | 48.43 | 51.57 |  |
| **Gender** |  |  |  | <0.001 |
| Male | 1966 (54.49) | 67.41 | 32.59 |  |
| Female | 3198 (45.51) | 76.09 | 23.91 |  |
| **Education** |  |  |  | 0.055 |
| No formal schooling | 2725 (41.01) | 64.24 | 35.76 |  |
| Up to primary | 776 (16.16) | 68.94 | 31.06 |  |
| Up to secondary | 1291 (34.73) | 81.79 | 18.21 |  |
| College and higher | 372 (8.10) | 67.5 | 32.5 |  |
| **Current smoking** |  |  |  | 0.0124 |
| Yes | 349 (10.52) | 77.99 | 22.01 |  |
| No | 4815 (89.48) | 70.58 | 29.42 |  |
| **Ever consumed alcohol** |  |  |  | <0.001 |
| Yes | 3125 (60.51) | 66.79 | 33.21 |  |
| No | 2039 (39.49) | 78.35 | 21.65 |  |
| **Sufficient fruit and vegetable intake** |  |  |  | 0.2175 |
| Yes | 1872 (36.98) | 72.71 | 27.29 |  |
| No | 3292 (63.02) | 70.56 | 29.44 |  |
| **Adequate physical activity** |  |  |  | 0.0223 |
| Yes | 878 (20.37) | 75.49 | 24.51 |  |
| No | 4286 (79.63) | 70.3 | 29.7 |  |
| **Diabetes** |  |  |  | <0.001 |
| No | 4999 (97.29) | 72.21 | 27.79 |  |
| Yes | 165 (2.71) | 40.85 | 59.15 |  |
| **BMI category** |  |  |  | <0.001 |
| Normal Weight | 1436 (32.52) | 81.91 | 18.09 |  |
| Under weight | 148 (3.55) | 91.59 | 8.41 |  |
| Overweight | 2064 (39.21) | 70.05 | 29.95 |  |
| Obesity | 1516 (24.72) | 56.64 | 43.36 |  |
| **Abdominal Obesity** |  |  |  | <0.001 |
| No | 2746 (62.75) | 77.04 | 22.96 |  |
| Yes | 2418 (37.25) | 61.79 | 38.21 |  |
| **Abdominal obesity, body mass index** |  |  |  | <0.001 |
| No, Normal | 1303 (30.54) | 82.18 | 17.82 |  |
| No, Underweight | 144 (3.49) | 91.89 | 8.11 |  |
| No , Overweight | 1126 (25.07) | 72.05 | 27.95 |  |
| No, Obesity | 173 (3.66) | 54.11 | 45.89 |  |
| Yes, Normal | 133 (1.98) | 77.73 | 22.27 |  |
| Yes, Underweight | 4 (0.06) | 74.76 | 25.24 |  |
| Yes, Overweight | 938 (14.15) | 66.5 | 33.5 |  |
| Yes, Obesity | 1343 (21.05) | 57.09 | 42.91 |  |

**Table 4: Distribution of the background characteristics of the participants in Nepal (n=4,894)**

| **Variables** | **Total (n= 4,894)** | **Normal BP** | **Hypertension** | p-value |
| --- | --- | --- | --- | --- |
|  | **Unweighted Frequency** | **Weighted Percentage** | **Weighted Percentage** |  |
| Weight in kg, mean (SD) | 56.07 (11.14) | 54.59 (10.04) | 60.10 (13.05) |  |
| Height in m, mean (SD) | 1.56 (0.08) | 1.56 (0.08) | 1.57 (0.09) |  |
| BMI in kg/m2, mean (SD) | 22.97 (4.08) | 22.47 (3.73) | 24.33 (4.71) |  |
| Systolic blood pressure in mmHg, mean (SD) | 125.16 (16.99) | 118.35 (10.31) | 143.76 (18.24) |  |
| Diastolic blood pressure in mmHg, mean (SD) | 82.15 (11.04) | 77.53 (7.21) | 94.79 (9.91) |  |
| **Age group** |  |  |  | <0.001 |
| 18-29 | 1096 (39.32) | 85.1 | 14.9 |  |
| 30-49 | 2339 (38.78) | 71.86 | 28.14 |  |
| 50-69 | 1459 (21.90) | 54.26 | 45.74 |  |
| **Gender** |  |  |  | <0.001 |
| Male | 1740 (47.07) | 67.63 | 32.37 |  |
| Female | 3154 (52.93) | 78.17 | 21.83 |  |
| **Education** |  |  |  | 0.055 |
| No formal schooling | 2067 (33.02) | 66.98 | 33.02 |  |
| Up to primary | 1381 (29.69) | 70.95 | 29.05 |  |
| Up to secondary | 1289 (33.88) | 81.04 | 18.96 |  |
| College and higher | 157 (3.41) | 75.37 | 24.63 |  |
| **Current smoking** |  |  |  | <0.003 |
| Yes | 961 (18.03) | 67.28 | 32.72 |  |
| No | 3933 (81.97) | 74.52 | 25.48 |  |
| **Ever consumed alcohol** |  |  |  | <0.001 |
| Yes | **1416 (29.43)** | 63.3 | 36.7 |  |
| No | 3478 (70.57) | 77.35 | 22.65 |  |
| **Sufficient fruit and vegetable intake** |  |  |  | 0.2034 |
| Yes | 509 (12.35) | 77.04 | 22.96 |  |
| No | 4385 (87.65) | 72.67 | 27.33 |  |
| **Adequate physical activity** |  |  |  | 0.9788 |
| Yes | 729 (15.65) | 73.28 | 26.72 |  |
| No | 4165 (84.35) | 73.2 | 26.8 |  |
| **Diabetes** |  |  |  | <0.001 |
| No | 4575 (93.96) | 74.4 | 25.6 |  |
| Yes | 319 (6.04) | 54.75 | 45.25 |  |
| **BMI category** |  |  |  | <0.001 |
| Normal Weight | 2158 (45.04) | 78.32 | 21.68 |  |
| Under weight | 441 (10.75) | 83.5 | 16.5 |  |
| Overweight | 1625 (32.04) | 70.18 | 29.82 |  |
| Obesity | 670 (12.16) | 53.17 | 46.83 |  |
| **Abdominal Obesity** |  |  |  | <0.001 |
| No | 3084 (69.87) | 77.88 | 22.12 |  |
| Yes | 1810 (30.13) | 62.39 | 37.61 |  |
| **Abdominal obesity, body mass index** |  |  |  | <0.001 |
| No, Normal | 1781 (38.89) | 79.19 | 20.81 |  |
| No, Underweight | 420 (10.54) | 83.55 | 16.45 |  |
| No , Overweight | 781 (18.01) | 72.7 | 27.3 |  |
| No, Obesity | 102 (2.44) | 70.68 | 29.32 |  |
| Yes, Normal | 377 (6.15) | 72.88 | 27.12 |  |
| Yes, Underweight | 21 (0.21) | 81.44 | 18.56 |  |
| Yes, Overweight | 844 (14.04) | 66.94 | 33.06 |  |
| Yes, Obesity | 568 (9.73) | 48.78 | 51.22 |  |

**Table 5: Distribution of the background characteristics of the participants in Sri Lanka (n=4,158)**

| **Variables** | **Total (n= 4,158)** | **Normal BP** | **Hypertension** | p-value |
| --- | --- | --- | --- | --- |
|  | **Unweighted Frequency** | **Weighted Percentage** | **Weighted Percentage** |  |
| Weight in kg, mean (SD) | 58.16 (14.87) | 57.13 (14.79) | 60.93 (14.17) |  |
| Height in m, mean (SD) | 1.60 (0.27) | 1.61 (0.25) | 1.59 (0.32) |  |
| BMI in kg/m2, mean (SD) | 22.86 (5.97) | 22.26 (5.92) | 24.49 (5.58) |  |
| Systolic blood pressure in mmHg, mean (SD) | 129.67 (44.91) | 117.70 (10.28) | 161.78 (88.38) |  |
| Diastolic blood pressure in mmHg, mean (SD) | 85.24 (44.60) | 76.19 (7.68) | 109.55 (87.91) |  |
| **Age group** |  |  |  | <0.001 |
| 18-29 | 600 (33.84) | 90.12 | 9.88 |  |
| 30-49 | 1976 (38.34) | 75.46 | 24.54 |  |
| 50-69 | 1582 (27.82) | 48.31 | 51.69 |  |
| **Gender** |  |  |  | 0.1074 |
| Male | 1615 (51.88) | 74.18 | 25.82 |  |
| Female | 2543 (48.12) | 71.45 | 28.55 |  |
| **Education** |  |  |  | 0.055 |
| No formal schooling | 213 (3.73) | 60.17 | 39.83 |  |
| Up to primary | 602 (11.28) | 65.89 | 34.11 |  |
| Up to secondary | 3169 (80.14) | 74.1 | 25.9 |  |
| College and higher | 174 (4.85) | 78.46 | 21.54 |  |
| **Current smoking** |  |  |  | 0.5198 |
| Yes | 513 (14.75) | 74.25 | 25.75 |  |
| No | 3645 (85.25) | 72.63 | 27.37 |  |
| **Ever consumed alcohol** |  |  |  | 0.0598 |
| Yes | 1150 (32.45) | 70.52 | 29.48 |  |
| No | 3008 (67.55) | 74 | 26 |  |
| **Sufficient fruit and vegetable intake** |  |  |  | 0.0194 |
| Yes | 2031 (48.65) | 70.72 | 29.28 |  |
| No | 2127 (51.35) | 74.9 | 25.1 |  |
| **Adequate physical activity** |  |  |  | 0.0472 |
| Yes | 315 (10.01) | 78.34 | 21.66 |  |
| No | 3843 (89.99) | 72.26 | 27.74 |  |
| **Diabetes** |  |  |  | <0.001 |
| No | 3636 (89.76) | 75.82 | 24.18 |  |
| Yes | 522 (10.24) | 46.95 | 53.05 |  |
| **BMI category** |  |  |  | <0.001 |
| Normal Weight | 1447 (37.76) | 80.5 | 19.5 |  |
| Under weight | 604 (16.65) | 82.79 | 17.21 |  |
| Overweight | 1416 (31.96) | 67.45 | 32.55 |  |
| Obesity | 691 (13.63) | 52.32 | 47.68 |  |
| **Abdominal Obesity** |  |  |  | <0.001 |
| No | 2250 (60.89) | 80.04 | 19.96 |  |
| Yes | 1908 (39.11) | 61.7 | 38.3 |  |
| **Abdominal obesity, body mass index** |  |  |  | <0.001 |
| No, Normal | 1118 (30.44) | 81.41 | 18.59 |  |
| No, Underweight | 566 (15.70) | 83.15 | 16.85 |  |
| No , Overweight | 495 (13.13) | 73.85 | 26.15 |  |
| No, Obesity | 71 (1.62) | 74.24 | 25.76 |  |
| Yes, Normal | 329 (7.31) | 76.68 | 23.32 |  |
| Yes, Underweight | 38 (0.95) | 76.84 | 23.16 |  |
| Yes, Overweight | 921 (18.83) | 62.99 | 37.01 |  |
| Yes, Obesity | 620 (12.02) | 49.37 | 50.63 |  |

**Table 6: Findings from the logistic regression models showing factors associated with hypertension in Afghanistan**

| **Variables** | **Crude Model** | | **Model 1** | | **Model 2** | | **Model 3** | | **Model 4** | |
| --- | --- | --- | --- | --- | --- | --- | --- | --- | --- | --- |
|  | **OR (95% CI)** | **p-value** | **OR (95% CI)** | **p-value** | **OR (95% CI)** | **p-value** | **OR (95% CI)** | **p-value** | **OR (95% CI)** | **p-value** |
| **Age group** |  |  |  |  |  |  |  |  |  |  |
| 18-29 | Ref |  | Ref |  | Ref |  | Ref |  | Ref |  |
| 30-49 | 1.67 (1.03-2.71) | 0.04 | 1.24 (0.77-1.98) | 0.37 | 1.16 (0.71-1.89) | 0.56 | 1.12 (0.69-1.81) | 0.65 | 1.12 (0.70-1.78) | 0.64 |
| 50-69 | 3.56 (2.09-6.06) | <0.001 | 2.70 (1.55-4.71) | <0.001 | 2.63 (1.51-4.57) | <0.001 | 2.59 (1.50-4.45) | <0.001 | 2.60 (1.55-4.37) | <0.001 |
| **Gender** |  |  |  |  |  |  |  |  |  |  |
| Male | Ref |  | Ref |  | Ref |  | Ref |  | Ref |  |
| Female | 1.64 (1.14-2.37) | 0.01 | 1.06 (0.67-1.67) | 0.81 | 1.26 (0.89-1.80) | 0.20 | 1.06 (0.70-1.62) | 0.77 | 1.06 (0.69-1.62) | 0.79 |
| **Education** |  |  |  |  |  |  |  |  |  |  |
| No formal schooling | Ref |  | Ref |  | Ref |  | Ref |  | Ref |  |
| Up to primary | 0.88 (0.65-1.19) | 0.39 | 1.09 (0.71-1.66) | 0.70 | 1.07 (0.71-1.61) | 0.76 | 1.06 (0.70-1.61) | 0.77 | 1.05 (0.70-1.58) | 0.80 |
| Up to secondary | 0.39 (0.25-0.59) | <0.001 | 0.63 (0.42-0.94) | 0.03 | 0.61 (0.43-0.86) | 0.01 | 0.63 (0.44-0.89) | 0.01 | 0.63 (0.44-0.91) | 0.01 |
| College and higher | 0.18 (0.08-0.40) | <0.001 | 0.18 (0.08-0.43) | <0.001 | 0.18 (0.08-0.43) | <0.001 | 0.18 (0.08-0.41) | <0.001 | 0.17 (0.07-0.40) | <0.001 |
| **Current smoking** |  |  |  |  |  |  |  |  |  |  |
| Yes | Ref |  | Ref |  | Ref |  | Ref |  | Ref |  |
| No | 1.24 (0.62-2.47) | 0.54 | 0.98 (0.54-1.80) | 0.96 | 0.90 (0.51-1.60) | 0.72 | 0.93 (0.53-1.65) | 0.81 | 0.93 (0.54-1.60) | 0.79 |
| **Alcohol use in last 30 days** |  |  |  |  |  |  |  |  |  |  |
| Yes | Ref |  | Ref |  | Ref |  | Ref |  | Ref |  |
| No | 0.44 (0.11-1.72) | 0.24 | 0.32 (0.09-1.08) | 0.07 | 0.28 (0.07-1.16) | 0.08 | 0.27 (0.07-1.08) | 0.06 | 0.27 (0.07-1.07) | 0.06 |
| **Sufficient fruit and vegetable intake** |  |  |  |  |  |  |  |  |  |  |
| Yes | Ref |  | Ref |  | Ref |  | Ref |  | Ref |  |
| No | 1.09 (0.65-1.82) | 0.74 | 0.93 (0.62-1.38) | 0.72 | 0.97 (0.68-1.40) | 0.88 | 0.99 (0.69-1.44) | 0.97 | 1.00 (0.70-1.43) | 1.00 |
| **Adequate physical activity** |  |  |  |  |  |  |  |  |  |  |
| Yes | Ref |  | Ref |  | Ref |  | Ref |  | Ref |  |
| No | 1.45 (0.90-2.33) | 0.12 | 1.00 (0.61-1.64) | 1.00 | 1.07 (0.66-1.72) | 0.79 | 1.04 (0.65-1.65) | 0.88 | 1.04 (0.68-1.60) | 0.85 |
| **Diabetes** |  |  |  |  |  |  |  |  |  |  |
| No | Ref |  | Ref |  | Ref |  | Ref |  | Ref |  |
| Yes | 4.61 (2.79-7.61) | <0.001 | 3.20 (1.92-5.31) | <0.001 | 3.16 (1.92-5.19) | <0.001 | 2.91 (1.79-4.73) | <0.001 | 2.99 (1.81-4.73) | <0.001 |
| **BMI category** |  |  |  |  |  |  |  |  |  |  |
| Normal Weight | Ref |  | Ref |  | Ref |  | Ref |  | Ref |  |
| Under weight | 0.54 (0.24-1.22) | 0.14 | - |  | 0.53 (0.23-1.19) | 0.12 | 0.56 (0.26-1.22) | 0.15 | - |  |
| Overweight | 1.93 (1.33-2.79) | <0.001 | - |  | 1.76 (1.23-2.70) | 0.01 | 1.39 (0.84-2.30) | 0.20 | - |  |
| Obesity | 5.85 (3.93-8.73) | <0.001 | - |  | 4.75 (5.23-7.30) | <0.001 | 3.25 (1.81-5.84) | <0.001 | - |  |
| **Abdominal Obesity** |  |  |  |  |  |  |  |  |  |  |
| No | Ref |  | Ref |  | Ref |  | Ref |  | Ref |  |
| Yes | 4.03 (3.09-5.27) | <0.001 | 3.36 (2.50-4.51) | <0.001 | - |  | 2.05 (1.27-3.31) | <0.001 | - |  |
| **Abdominal obesity, body mass index** |  |  |  |  |  |  |  |  |  |  |
| No, Normal | Ref |  | Ref |  | Ref |  | Ref |  | Ref |  |
| No, Underweight | 0.48 (0.22-1.02) | 0.06 | - |  | - |  | - |  | 0.49 (0.25-0.95) | 0.03 |
| No , Overweight | 1.56 (0.75-3.27) | 0.23 | - |  | - |  | - |  | 1.59 (0.68-3.70) | 0.28 |
| No, Obesity | 3.13 (1.55-6.35) | <0.001 | - |  | - |  | - |  | 2.80 (1.48-5.30) | <0.001 |
| Yes, Normal | 2.40 (1.07-5.38) | 0.03 | - |  | - |  | - |  | 2.18 (0.94-5.09) | 0.07 |
| Yes, Underweight | 1.76 (0.36-8.71) | 0.49 | - |  | - |  | - |  | 1.57 (0.29-8.69) | 0.60 |
| Yes, Overweight | 3.29 (2.16-5.01) | <0.001 | - |  | - |  | - |  | 2.75 (1.75-4.34) | <0.001 |
| Yes, Obesity | 8.41 (5.85-12.09) | <0.001 | - |  | - |  | - |  | 6.94 (4.68-10.30) | <0.001 |

**Table 7: Findings from the logistic regression models showing factors associated with hypertension in Bangladesh**

| **Variables** | **Crude Model** | | **Model 1** | | **Model 2** | | **Model 3** | | **Model 4** | |
| --- | --- | --- | --- | --- | --- | --- | --- | --- | --- | --- |
|  | **OR (95% CI)** | **p-value** | **OR (95% CI)** | **p-value** | **OR (95% CI)** | **p-value** | **OR (95% CI)** | **p-value** | **OR (95% CI)** | **p-value** |
| **Age group** |  |  |  |  |  |  |  |  |  |  |
| 18-29 | Ref |  | Ref |  | Ref |  | Ref |  | Ref |  |
| 30-49 | 2.83 (2.21-3.64) | <0.001 | 2.64 (2.00-3.48) | <0.001 | 2.67 (2.01-3.53) | <0.001 | 2.55 (1.93-3.38) | <0.001 | 2.61 (1.97-3.45) | <0.001 |
| 50-69 | 5.82 (4.54-7.48) | <0.001 | 6.00 (4.50-7.99) | <0.001 | 6.74 (5.03-9.04) | <0.001 | 6.31 (4.72-8.43) | <0.001 | 6.43 (4.81-8.58) | <0.001 |
| **Gender** |  |  |  |  |  |  |  |  |  |  |
| Male | Ref |  | Ref |  | Ref |  | Ref |  | Ref |  |
| Female | 1.66 (1.40-1.97) | <0.001 | 1.14 (0.92-1.42) | 0.23 | 1.30 (1.04-1.63) | 0.02 | 1.20 (0.96-1.49) | 0.11 | 1.18 (0.95-1.48) | 0.13 |
| **Education** |  |  |  |  |  |  |  |  |  |  |
| No formal schooling | Ref |  | Ref |  | Ref |  | Ref |  | Ref |  |
| Up to primary | 0.77 (0.63-0.94) | 0.01 | 1.22 (0.99-1.50) | 0.06 | 1.18 (0.96-1.45) | 0.11 | 1.15 (0.94-1.42) | 0.17 | 1.15 (0.94-1.42) | 0.18 |
| Up to secondary | 0.79 (0.62-1.02) | 0.07 | 1.46 (1.10-1.95) | 0.01 | 1.42 (1.08-1.88) | 0.01 | 1.36 (1.02-1.80) | 0.04 | 1.37 (1.03-1.82) | 0.03 |
| College and higher | 0.79 (0.54-1.15) | 0.22 | 1.19 (0.79-1.80) | 0.40 | 1.11 (0.74-1.65) | 0.61 | 1.07 (0.71-1.61) | 0.74 | 1.09 (0.73-1.64) | 0.67 |
| **Current smoking** |  |  |  |  |  |  |  |  |  |  |
| Yes | Ref |  | Ref |  | Ref |  | Ref |  | Ref |  |
| No | 1.91 (1.46-2.49) | <0.001 | 1.65 (1.18-2.31) | <0.001 | 1.54 (1.11-2.14) | 0.01 | 1.54 (1.11-2.15) | 0.01 | 1.53 (1.10-2.12) | 0.01 |
| **Alcohol use in last 30 days** |  |  |  |  |  |  |  |  |  |  |
| Yes | Ref |  | Ref |  | Ref |  | Ref |  | Ref |  |
| No | 1.86 (1.24-2.78) | <0.001 | 0.96 (0.61-1.49) | 0.84 | 0.98 (0.62-1.55) | 0.94 | 0.98 (0.62-1.54) | 0.93 | 0.99 (0.63-1.56) | 0.97 |
| **Sufficient fruit and vegetable intake** |  |  |  |  |  |  |  |  |  |  |
| Yes | Ref |  | Ref |  | Ref |  | Ref |  | Ref |  |
| No | 1.23 (1.00-1.51) | 0.05 | 1.22 (0.96-1.54) | 0.11 | 1.20 (0.95-1.52) | 0.13 | 1.23 (0.97-1.57) | 0.09 | 1.25 (0.99-1.59) | 0.07 |
| **Adequate physical activity** |  |  |  |  |  |  |  |  |  |  |
| Yes | Ref |  | Ref |  | Ref |  | Ref |  | Ref |  |
| No | 1.81 (1.41-2.33) | <0.001 | 1.23 (0.95-1.61) | 0.12 | 1.24 (0.95-1.61) | 0.11 | 1.24 (0.95-1.61) | 0.11 | 1.24 (0.95-1.62) | 0.11 |
| **Diabetes** |  |  |  |  |  |  |  |  |  |  |
| No | Ref |  | Ref |  | Ref |  | Ref |  | Ref |  |
| Yes | 4.11 (3.02-5.59) | <0.001 | 2.33 (1.71-3.17) | <0.001 | 2.29 (1.68-3.11) | <0.001 | 2.18 (1.59-3.00) | <0.001 | 2.18 (1.59-2.99) | <0.001 |
| **BMI category** |  |  |  |  |  |  |  |  |  |  |
| Normal Weight | Ref |  | Ref |  | Ref |  | Ref |  | Ref |  |
| Under weight | 0.54 (0.39-0.75) | <0.001 | - |  | 0.50 (0.36-0.70) | <0.001 | 0.51 (0.36-0.72) | <0.001 | - |  |
| Overweight | 2.21 (1.79-2.73) | <0.001 | - |  | 1.95 (1.55-2.46) | <0.001 | 1.62 (1.24-2.12) | <0.001 | - |  |
| Obesity | 3.42 (2.61-4.49) | <0.001 | - |  | 2.61 (1.99-3.43) | <0.001 | 1.83 (1.31-2.57) | <0.001 | - |  |
| **Abdominal Obesity** |  |  |  |  |  |  |  |  |  |  |
| No | Ref |  | Ref |  | Ref |  | Ref |  | Ref |  |
| Yes | 3.45 (2.85-4.19) | <0.001 | 2.43 (1.96-3.02) | <0.001 | - |  | 1.55 (1.18-2.04) | <0.001 | - |  |
| **Abdominal obesity, body mass index** |  |  |  |  |  |  |  |  |  |  |
| No, Normal | Ref |  | Ref |  | Ref |  | Ref |  | Ref |  |
| No, Underweight | 0.60 (0.43-0.86) | 0.01 | - |  | - |  | - |  | 0.56 (0.39-0.81) | <0.001 |
| No , Overweight | 1.74 (1.29-2.35) | <0.001 | - |  | - |  | - |  | 1.86 (1.37-2.52) | <0.001 |
| No, Obesity | 2.41 (1.13-5.14) | 0.02 | - |  | - |  | - |  | 3.19 (1.40-7.31) | 0.01 |
| Yes, Normal | 4.13 (2.20-7.78) | <0.001 | - |  | - |  | - |  | 3.18 (1.83-5.54) | <0.001 |
| Yes, Underweight | 0.67 (0.10-4.35) | 0.68 | - |  | - |  | - |  | 0.27 (0.03-2.12) | 0.21 |
| Yes, Overweight | 3.52 (2.74-4.54) | <0.001 | - |  | - |  | - |  | 2.53 (1.90-3.37) | <0.001 |
| Yes, Obesity | 3.98 (2.94-5.38) | <0.001 | - |  | - |  | - |  | 2.95 (2.19-3.97) | <0.001 |

**Table 8: Findings from the logistic regression models showing factors associated with hypertension in Bhutan**

| **Variables** | **Crude Model** | | **Model 1** | | **Model 2** | | **Model 3** | | **Model 4** | |
| --- | --- | --- | --- | --- | --- | --- | --- | --- | --- | --- |
|  | **OR (95% CI)** | **p-value** | **OR (95% CI)** | **p-value** | **OR (95% CI)** | **p-value** | **OR (95% CI)** | **p-value** | **OR (95% CI)** | **p-value** |
| **Age group** |  |  |  |  |  |  |  |  |  |  |
| 18-29 | Ref |  | Ref |  | Ref |  | Ref |  | Ref |  |
| 30-49 | 4.25 (3.30-5.47) | <0.001 | 3.26 (2.39-4.44) | <0.001 | 2.93 (2.11-4.08) | <0.001 | 2.92 (2.10-4.05) | <0.001 | 2.89 (2.09-4.01) | <0.001 |
| 50-69 | 8.78 (6.23-12.39) | <0.001 | 6.32 (4.19-9.54) | <0.001 | 6.33 (4.19-9.58) | <0.001 | 6.14 (4.07-9.26) | <0.001 | 6.08 (4.05-9.13) | <0.001 |
| **Gender** |  |  |  |  |  |  |  |  |  |  |
| Male | Ref |  | Ref |  | Ref |  | Ref |  | Ref |  |
| Female | 0.65 (0.54-0.78) | <0.001 | 0.52 (0.42-0.64) | <0.001 | 0.59 (0.48-0.73) | <0.001 | 0.55 (0.44-0.68) | <0.001 | 0.54 (0.44-0.67) | <0.001 |
| **Education** |  |  |  |  |  |  |  |  |  |  |
| No formal schooling | Ref |  | Ref |  | Ref |  | Ref |  | Ref |  |
| Up to primary | 0.81 (0.63-1.04) | 0.10 | 0.98 (0.73-1.33) | 0.92 | 1.00 (0.72-1.37) | 0.98 | 0.98 (0.71-1.35) | 0.91 | 0.98 (0.72-1.35) | 0.92 |
| Up to secondary | 0.40 (0.32-0.50) | <0.001 | 0.84 (0.63-1.11) | 0.22 | 0.82 (0.63-1.08) | 0.15 | 0.82 (0.62-1.08) | 0.16 | 0.81 (0.62-1.07) | 0.14 |
| College and higher | 0.86 (0.69-1.09) | 0.21 | 0.97 (0.73-1.30) | 0.86 | 1.00 (0.76-1.31) | 1.00 | 0.97 (0.74-1.28) | 0.84 | 0.97 (0.73-1.27) | 0.81 |
| **Current smoking** |  |  |  |  |  |  |  |  |  |  |
| Yes | Ref |  | Ref |  | Ref |  | Ref |  | Ref |  |
| No | 1.48 (1.09-2.00) | 0.01 | 1.18 (0.88-1.59) | 0.27 | 1.18 (0.86-1.64) | 0.30 | 1.17 (0.85-1.60) | 0.34 | 1.16 (0.84-1.61) | 0.37 |
| **Alcohol use in last 30 days** |  |  |  |  |  |  |  |  |  |  |
| Yes | Ref |  | Ref |  | Ref |  | Ref |  | Ref |  |
| No | 0.56 (0.45-0.68) | <0.001 | 0.64 (0.52-0.80) | <0.001 | 0.65 (0.53-0.82) | <0.001 | 0.66 (0.53-0.82) | <0.001 | 0.65 (0.52-0.82) | <0.001 |
| **Sufficient fruit and vegetable intake** |  |  |  |  |  |  |  |  |  |  |
| Yes | Ref |  | Ref |  | Ref |  | Ref |  | Ref |  |
| No | 1.11 (0.94-1.32) | 0.22 | 1.11 (0.92-1.33) | 0.27 | 1.12 (0.93-1.34) | 0.22 | 1.12 (0.93-1.34) | 0.22 | 1.12 (0.93-1.34) | 0.22 |
| **Adequate physical activity** |  |  |  |  |  |  |  |  |  |  |
| Yes | Ref |  | Ref |  | Ref |  | Ref |  | Ref |  |
| No | 1.30 (1.04-1.63) | 0.02 | 1.11 (0.88-1.40) | 0.39 | 1.11 (0.88-1.40) | 0.38 | 1.11 (0.88-1.41) | 0.36 | 1.11 (0.88-1.41) | 0.36 |
| **Diabetes** |  |  |  |  |  |  |  |  |  |  |
| No | Ref |  | Ref |  | Ref |  | Ref |  | Ref |  |
| Yes | 3.76 (2.31-6.13) | <0.001 | 2.02 (1.21-3.39) | 0.01 | 1.90 (1.11-3.25) | 0.02 | 1.88 (1.11-3.20) | 0.02 | 1.90 (1.12-3.20) | 0.02 |
| **BMI category** |  |  |  |  |  |  |  |  |  |  |
| Normal Weight | Ref |  | Ref |  | Ref |  | Ref |  | Ref |  |
| Under weight | 0.42 (0.23-0.74) | <0.001 | - |  | 0.55 (0.31-0.96) | 0.04 | 0.56 (0.32-0.99) | 0.05 | - |  |
| Over weight/Obesity | 1.94 (1.51-2.48) | <0.001 | - |  | 1.72 (1.32-2.25) | <0.001 | 1.60 (1.21-2.12) | <0.001 | - |  |
| Obesity | 3.47 (2.68-4.48) | <0.001 | - |  | 2.86 (2.14-3.82) | <0.001 | 2.36 (1.67-3.33) | <0.001 | - |  |
| **Abdominal Obesity** |  |  |  |  |  |  |  |  |  |  |
| No | Ref |  | Ref |  | Ref |  | Ref |  | Ref |  |
| Yes | 2.07 (1.74-2.47) | <0.001 | 2.02 (1.66-2.46) | <0.001 | - |  | 1.31 (1.03-1.66) | 0.03 | - |  |
| **Abdominal obesity, body mass index** |  |  |  |  |  |  |  |  |  |  |
| No, Normal | Ref |  | Ref |  | Ref |  | Ref |  | Ref |  |
| No, Underweight | 0.41 (0.22-0.74) | <0.001 | - |  | - |  | - |  | 0.56 (0.32-0.99) | 0.05 |
| No , Overweight | 1.79 (1.36-2.35) | <0.001 | - |  | - |  | - |  | 1.58 (1.16-2.15) | <0.001 |
| No, Obesity | 3.91 (2.37-6.45) | <0.001 | - |  | - |  | - |  | 2.95 (1.78-4.90) | <0.001 |
| Yes, Normal | 1.32 (0.82-2.13) | 0.25 | - |  | - |  | - |  | 1.58 (0.95-2.61) | 0.08 |
| Yes, Underweight | 1.56 (0.15-16.00) | 0.71 | - |  | - |  | - |  | 1.15 (0.14-9.31) | 0.90 |
| Yes, Overweight | 2.32 (1.74-3.10) | <0.001 | - |  | - |  | - |  | 2.22 (1.64-3.00) | <0.001 |
| Yes, Obesity | 3.47 (2.68-4.49) | <0.001 | - |  | - |  | - |  | 3.02 (2.23-4.09) | <0.001 |

**Table 9: Findings from the logistic regression models showing factors associated with hypertension in Nepal**

| **Variables** | **Crude Model** | | **Model 1** | | **Model 2** | | **Model 3** | | **Model 4** | |
| --- | --- | --- | --- | --- | --- | --- | --- | --- | --- | --- |
|  | **OR (95% CI)** | **p-value** | **OR (95% CI)** | **p-value** | **OR (95% CI)** | **p-value** | **OR (95% CI)** | **p-value** | **OR (95% CI)** | **p-value** |
| **Age group** |  |  |  |  |  |  |  |  |  |  |
| 18-29 | Ref |  | Ref |  | Ref |  | Ref |  | Ref |  |
| 30-49 | 2.24 (1.70-2.94) | <0.001 | 1.67 (1.26-2.21) | <0.001 | 1.70 (1.27-2.28) | <0.001 | 1.60 (1.20-2.14) | <0.001 | 1.60 (1.20-2.14) | <0.001 |
| 50-69 | 4.81 (3.65-6.35) | <0.001 | 3.49 (2.47-4.94) | <0.001 | 3.80 (2.65-5.44) | <0.001 | 3.56 (2.50-5.08) | <0.001 | 3.58 (2.51-5.11) | <0.001 |
| **Gender** |  |  |  |  |  |  |  |  |  |  |
| Male | Ref |  | Ref |  | Ref |  | Ref |  | Ref |  |
| Female | 0.58 (0.48-0.71) | <0.001 | 0.48 (0.37-0.63) | <0.001 | 0.59 (0.46-0.77) | <0.001 | 0.52 (0.39-0.67) | <0.001 | 0.52 (0.39-0.68) | <0.001 |
| **Education** |  |  |  |  |  |  |  |  |  |  |
| No formal schooling | Ref |  | Ref |  | Ref |  | Ref |  | Ref |  |
| Up to primary | 0.83 (0.68-1.02) | 0.07 | 0.96 (0.75-1.21) | 0.72 | 0.95 (0.73-1.22) | 0.67 | 0.94 (0.73-1.20) | 0.61 | 0.93 (0.72-1.19) | 0.56 |
| Up to secondary | 0.47 (0.36-0.63) | <0.001 | 0.71 (0.50-0.99) | 0.05 | 0.68 (0.48-0.96) | 0.03 | 0.67 (0.48-0.94) | 0.02 | 0.67 (0.48-0.94) | 0.02 |
| College and higher | 0.66 (0.35-1.25) | 0.20 | 0.83 (0.41-1.69) | 0.61 | 0.78 (0.41-1.49) | 0.45 | 0.77 (0.39-1.50) | 0.44 | 0.78 (0.39-1.56) | 0.48 |
| **Current smoking** |  |  |  |  |  |  |  |  |  |  |
| Yes | Ref |  | Ref |  | Ref |  | Ref |  | Ref |  |
| No | 0.70 (0.56-0.89) | <0.001 | 1.15 (0.90-1.48) | 0.26 | 1.15 (0.89-1.49) | 0.29 | 1.13 (0.88-1.46) | 0.34 | 1.12 (0.87-1.44) | 0.40 |
| **Alcohol use in last 30 days** |  |  |  |  |  |  |  |  |  |  |
| Yes | Ref |  | Ref |  | Ref |  | Ref |  | Ref |  |
| No | 0.51 (0.41-0.63) | <0.001 | 0.62 (0.48-0.79) | <0.001 | 0.63 (0.48-0.81) | <0.001 | 0.63 (0.49-0.81) | <0.001 | 0.63 (0.49-0.81) | <0.001 |
| **Sufficient fruit and vegetable intake** |  |  |  |  |  |  |  |  |  |  |
| Yes | Ref |  | Ref |  | Ref |  | Ref |  | Ref |  |
| No | 1.26 (0.88-1.81) | 0.20 | 1.19 (0.86-1.66) | 0.30 | 1.23 (0.89-1.71) | 0.21 | 1.21 (0.87-1.68) | 0.25 | 1.22 (0.88-1.69) | 0.23 |
| **Adequate physical activity** |  |  |  |  |  |  |  |  |  |  |
| Yes | Ref |  | Ref |  | Ref |  | Ref |  | Ref |  |
| No | 1.00 (0.75-1.35) | 0.98 | 0.95 (0.70-1.30) | 0.76 | 0.92 (0.68-1.24) | 0.58 | 0.92 (0.68-1.25) | 0.60 | 0.93 (0.69-1.26) | 0.64 |
| **Diabetes** |  |  |  |  |  |  |  |  |  |  |
| No | Ref |  | Ref |  | Ref |  | Ref |  | Ref |  |
| Yes | 2.40 (1.69-3.42) | <0.001 | 1.75 (1.27-2.41) | <0.001 | 1.77 (1.28-2.44) | <0.001 | 1.71 (1.25-2.35) | <0.001 | 1.70 (1.24-2.33) | <0.001 |
| **BMI category** |  |  |  |  |  |  |  |  |  |  |
| Normal Weight | Ref |  | Ref |  | Ref |  | Ref |  | Ref |  |
| Under weight | 0.71 (0.47-1.09) | 0.12 | - |  | 0.67 (0.43-1.05) | 0.08 | 0.72 (0.46-1.13) | 0.16 | - |  |
| Over weight/Obesity | 1.54 (1.21-1.95) | <0.001 | - |  | 1.53 (1.17-1.99) | <0.001 | 1.32 (0.99-1.76) | 0.06 | - |  |
| Obesity | 3.18 (2.40-4.22) | <0.001 | - |  | 3.24 (2.37-4.43) | <0.001 | 2.35 (1.66-3.32) | <0.001 | - |  |
| **Abdominal Obesity** |  |  |  |  |  |  |  |  |  |  |
| No | Ref |  | Ref |  | Ref |  | Ref |  | Ref |  |
| Yes | 2.12 (1.74-2.59) | <0.001 | 2.43 (1.95-3.02) | <0.001 | - |  | 1.69 (1.31-2.18) | <0.001 | - |  |
| **Abdominal obesity, body mass index** |  |  |  |  |  |  |  |  |  |  |
| No, Normal | Ref |  | Ref |  | Ref |  | Ref |  | Ref |  |
| No, Underweight | 0.75 (0.48-1.17) | 0.20 | - |  | - |  | - |  | 0.71 (0.45-1.14) | 0.16 |
| No , Overweight | 1.43 (1.01-2.01) | 0.04 | - |  | - |  | - |  | 1.36 (0.94-1.96) | 0.10 |
| No, Obesity | 1.58 (0.85-2.92) | 0.14 | - |  | - |  | - |  | 1.38 (0.72-2.64) | 0.33 |
| Yes, Normal | 1.42 (0.94-2.12) | 0.09 | - |  | - |  | - |  | 1.54 (1.03-2.28) | 0.03 |
| Yes, Underweight | 0.87 (0.23-3.25) | 0.83 | - |  | - |  | - |  | 0.91 (0.22-3.69) | 0.89 |
| Yes, Overweight | 1.88 (1.44-2.45) | <0.001 | - |  | - |  | - |  | 2.08 (1.54-2.81) | <0.001 |
| Yes, Obesity | 3.99 (2.91-5.48) | <0.001 | - |  | - |  | - |  | 4.40 (3.05-6.34) | <0.001 |

**Table 10: Findings from the logistic regression models showing factors associated with hypertension in Sri Lanka**

| **Variables** | **Crude Model** | | **Model 1** | | **Model 2** | | **Model 3** | | **Model 4** | |
| --- | --- | --- | --- | --- | --- | --- | --- | --- | --- | --- |
|  | **OR (95% CI)** | **p-value** | **OR (95% CI)** | **p-value** | **OR (95% CI)** | **p-value** | **OR (95% CI)** | **p-value** | **OR (95% CI)** | **p-value** |
| **Age group** |  |  |  |  |  |  |  |  |  |  |
| 18-29 | Ref |  | Ref |  | Ref |  | Ref |  | Ref |  |
| 30-49 | 2.97 (2.10-4.20) | <0.001 | 2.46 (1.74-3.48) | <0.001 | 2.30 (1.61-3.29) | <0.001 | 2.26 (1.59-3.22) | <0.001 | 2.26 (1.59-3.21) | <0.001 |
| 50-69 | 9.75 (6.91-13.76) | <0.001 | 7.96 (5.62-11.29) | <0.001 | 7.80 (5.46-11.13) | <0.001 | 7.63 (5.36-10.85) | <0.001 | 7.60 (5.35-10.81) | <0.001 |
| **Gender** |  |  |  |  |  |  |  |  |  |  |
| Male | Ref |  | Ref |  | Ref |  | Ref |  | Ref |  |
| Female | 1.14 (0.97-1.35) | 0.12 | 0.86 (0.66-1.12) | 0.26 | 1.01 (0.78-1.30) | 0.97 | 0.89 (0.68-1.18) | 0.42 | 0.90 (0.69-1.18) | 0.43 |
| **Education** |  |  |  |  |  |  |  |  |  |  |
| No formal schooling | Ref |  | Ref |  | Ref |  | Ref |  | Ref |  |
| Up to primary | 0.78 (0.53-1.15) | 0.21 | 0.73 (0.50-1.08) | 0.12 | 0.75 (0.50-1.12) | 0.15 | 0.75 (0.50-1.11) | 0.15 | 0.75 (0.50-1.12) | 0.16 |
| Up to secondary | 0.53 (0.38-0.75) | <0.001 | 0.87 (0.62-1.22) | 0.43 | 0.86 (0.60-1.24) | 0.42 | 0.85 (0.59-1.22) | 0.37 | 0.85 (0.60-1.22) | 0.39 |
| College and higher | 0.42 (0.25-0.71) | <0.001 | 0.70 (0.42-1.17) | 0.17 | 0.67 (0.39-1.15) | 0.14 | 0.66 (0.38-1.12) | 0.12 | 0.66 (0.39-1.14) | 0.14 |
| **Current smoking** |  |  |  |  |  |  |  |  |  |  |
| Yes | Ref |  | Ref |  | Ref |  | Ref |  | Ref |  |
| No | 1.10 (0.86-1.41) | 0.46 | 1.09 (0.80-1.49) | 0.59 | 1.05 (0.77-1.45) | 0.75 | 1.04 (0.76-1.43) | 0.81 | 1.04 (0.76-1.43) | 0.81 |
| **Alcohol use in last 30 days** |  |  |  |  |  |  |  |  |  |  |
| Yes | Ref |  | Ref |  | Ref |  | Ref |  | Ref |  |
| No | 0.85 (0.71-1.01) | 0.07 | 0.87 (0.67-1.13) | 0.28 | 0.87 (0.67-1.14) | 0.31 | 0.87 (0.67-1.13) | 0.30 | 0.87 (0.67-1.14) | 0.32 |
| **Sufficient fruit and vegetable intake** |  |  |  |  |  |  |  |  |  |  |
| Yes | Ref |  | Ref |  | Ref |  | Ref |  | Ref |  |
| No | 0.81 (0.68-0.97) | 0.02 | 0.84 (0.70-1.01) | 0.06 | 0.85 (0.71-1.03) | 0.10 | 0.85 (0.71-1.03) | 0.09 | 0.85 (0.71-1.03) | 0.09 |
| **Adequate physical activity** |  |  |  |  |  |  |  |  |  |  |
| Yes | Ref |  | Ref |  | Ref |  | Ref |  | Ref |  |
| No | 1.38 (1.00-1.90) | 0.05 | 1.01 (0.70-1.46) | 0.96 | 1.01 (0.70-1.46) | 0.95 | 1.01 (0.70-1.45) | 0.98 | 1.00 (0.69-1.45) | 0.99 |
| **Diabetes** |  |  |  |  |  |  |  |  |  |  |
| No | Ref |  | Ref |  | Ref |  | Ref |  | Ref |  |
| Yes | 3.53 (2.80-4.45) | <0.001 | 1.99 (1.58-2.51) | <0.001 | 1.98 (1.57-2.50) | <0.001 | 1.94 (1.53-2.44) | <0.001 | 1.94 (1.54-2.46) | <0.001 |
| **BMI category** |  |  |  |  |  |  |  |  |  |  |
| Normal Weight | Ref |  | Ref |  | Ref |  | Ref |  | Ref |  |
| Under weight | 0.85 (0.65-1.13) | 0.27 | - |  | 0.97 (0.72-1.31) | 0.86 | 1.03 (0.76-1.40) | 0.84 | - |  |
| Over weight/Obesity | 1.99 (1.64-2.41) | <0.001 | - |  | 1.80 (1.47-2.20) | <0.001 | 1.52 (1.23-1.89) | <0.001 | - |  |
| Obesity | 3.72 (2.99-4.63) | <0.001 | - |  | 3.31 (2.56-4.29) | <0.001 | 2.55 (1.93-3.38) | <0.001 | - |  |
| **Abdominal Obesity** |  |  |  |  |  |  |  |  |  |  |
| No | Ref |  | Ref |  | Ref |  | Ref |  | Ref |  |
| Yes | 2.48 (2.10-2.93) | <0.001 | 2.24 (1.84-2.72) | <0.001 | - |  | 1.55 (1.23-1.95) | <0.001 | - |  |
| **Abdominal obesity, body mass index** |  |  |  |  |  |  |  |  |  |  |
| No, Normal | Ref |  | Ref |  | Ref |  | Ref |  | Ref |  |
| No, Underweight | 0.88 (0.65-1.20) | 0.43 | - |  | - |  | - |  | 1.00 (0.72-1.39) | 0.99 |
| No , Overweight | 1.55 (1.18-2.03) | <0.001 | - |  | - |  | - |  | 1.43 (1.07-1.92) | 0.02 |
| No, Obesity | 1.53 (0.83-2.83) | 0.17 | - |  | - |  | - |  | 1.69 (0.92-3.12) | 0.09 |
| Yes, Normal | 1.33 (0.95-1.87) | 0.10 | - |  | - |  | - |  | 1.30 (0.90-1.87) | 0.16 |
| Yes, Underweight | 1.34 (0.61-2.93) | 0.47 | - |  | - |  | - |  | 1.44 (0.66-3.16) | 0.36 |
| Yes, Overweight | 2.56 (2.01-3.26) | <0.001 | - |  | - |  | - |  | 2.29 (1.77-2.98) | <0.001 |
| Yes, Obesity | 4.43 (3.47-5.67) | <0.001 | - |  | - |  | - |  | 3.96 (2.94-5.32) | <0.001 |

**Table 11: Findings from the logistic regression models showing factors associated with hypertension in Afghanistan**

| **Variables** | **Crude Model** | | **Model 1** | | **Model 2** | | **Model 3** | | **Model 4** | |
| --- | --- | --- | --- | --- | --- | --- | --- | --- | --- | --- |
|  | **OR (95% CI)** | **p-value** | **OR (95% CI)** | **p-value** | **OR (95% CI)** | **p-value** | **OR (95% CI)** | **p-value** | **OR (95% CI)** | **p-value** |
| **Age group** |  |  |  |  |  |  |  |  |  |  |
| 18-29 | Ref |  | Ref |  | Ref |  | Ref |  | Ref |  |
| 30-49 | 1.67 (1.03-2.71) | 0.04 | 1.33 (0.83 - 2.15) | 0.24 | 1.16 (0.71-1.89) | 0.56 | 1.13 (0.70 - 1.84) | 0.61 | 1.14 (0.70 - 1.87) | 0.60 |
| 50-69 | 3.56 (2.09-6.06) | <0.001 | 2.76 (1.58 - 4.82) | <0.001 | 2.63 (1.51-4.57) | <0.001 | 2.57 (1.50 - 4.42) | <0.001 | 2.59 (1.50 - 4.47) | 0<0.001 |
| **Gender** |  |  |  |  |  |  |  |  |  |  |
| Male | Ref |  | Ref |  | Ref |  | Ref |  | Ref |  |
| Female | 1.64 (1.14-2.37) | 0.01 | 1.41 (0.91 - 2.17) | 0.13 | 1.26 (0.89-1.80) | 0.20 | 1.21 (0.83 - 1.77) | 0.31 | 1.21 (0.84 - 1.77) | 0.31 |
| **Education** |  |  |  |  |  |  |  |  |  |  |
| No formal schooling | Ref |  | Ref |  | Ref |  | Ref |  | Ref |  |
| Up to primary | 0.88 (0.65-1.19) | 0.39 | 1.08 (0.72 - 1.61) | 0.71 | 1.07 (0.71-1.61) | 0.76 | 1.06 (0.70 - 1.59) | 0.79 | 1.05 (0.70 - 1.55) | 0.82 |
| Up to secondary | 0.39 (0.25-0.59) | <0.001 | 0.64 (0.43 - 0.95) | 0.03 | 0.61 (0.43-0.86) | 0.01 | 0.62 (0.44 - 0.87) | 0.01 | 0.62 (0.44 - 0.88) | 0.01 |
| College and higher | 0.18 (0.08-0.40) | <0.001 | 0.21 (0.09 - 0.47) | <0.001 | 0.18 (0.08-0.43) | <0.001 | 0.18 (0.08 - 0.42) | <0.001 | 0.18 (0.08 - 0.41) | <0.001 |
| **Current smoking** |  |  |  |  |  |  |  |  |  |  |
| Yes | Ref |  | Ref |  | Ref |  | Ref |  | Ref |  |
| No | 1.24 (0.62-2.47) | 0.54 | 0.88 (0.50 - 1.52) | 0.64 | 0.90 (0.51-1.60) | 0.72 | 0.90 (0.51 - 1.58) | 0.70 | 0.88 (0.50 - 1.55) | 0.67 |
| **Alcohol use in last 30 days** |  |  |  |  |  |  |  |  |  |  |
| Yes | Ref |  | Ref |  | Ref |  | Ref |  | Ref |  |
| No | 0.44 (0.11-1.72) | 0.24 | 0.30 (0.09 - 1.04) | 0.06 | 0.28 (0.07-1.16) | 0.08 | 0.26 (0.06 - 1.02) | 0.05 | 0.26 (0.06 - 1.04) | 0.06 |
| **Sufficient fruit and vegetable intake** |  |  |  |  |  |  |  |  |  |  |
| Yes | Ref |  | Ref |  | Ref |  | Ref |  | Ref |  |
| No | 1.09 (0.65-1.82) | 0.74 | 0.89 (0.59 - 1.36) | 0.59 | 0.97 (0.68-1.40) | 0.88 | 0.98 (0.68 - 1.43) | 0.93 | 0.98 (0.67 - 1.43) | 0.92 |
| **Adequate physical activity** |  |  |  |  |  |  |  |  |  |  |
| Yes | Ref |  | Ref |  | Ref |  | Ref |  | Ref |  |
| No | 1.45 (0.90-2.33) | 0.12 | 1.01 (0.61 - 1.67) | 0.97 | 1.07 (0.66-1.72) | 0.79 | 1.06 (0.66 - 1.69) | 0.82 | 1.06 (0.67 - 1.69) | 0.80 |
| **Diabetes** |  |  |  |  |  |  |  |  |  |  |
| No | Ref |  | Ref |  | Ref |  | Ref |  | Ref |  |
| Yes | 4.61 (2.79-7.61) | <0.001 | 4.05 (2.33 - 7.02) | 0.00 | 3.16 (1.92-5.19) | <0.001 | 3.24 (1.93 - 5.42) | 0.00 | 3.23 (1.93 - 5.40) | <0.001 |
| **BMI category** |  |  |  |  |  |  |  |  |  |  |
| Normal Weight | Ref |  | Ref |  | Ref |  | Ref |  | Ref |  |
| Under weight | 0.54 (0.24 - 1.22) | 0.14 | - |  | 0.53 (0.23-1.19) | 0.12 | 0.60 (0.27 - 1.36) | 0.22 | - |  |
| Overweight | 1.93 (1.33 - 2.79) | <0.001 | - |  | 1.76 (1.23-2.70) | 0.01 | 1.55 (1.02 - 2.36) | 0.04 | - |  |
| Obesity | 5.85 (3.93 - 8.73) | <0.001 | - |  | 4.75 (5.23-7.30) | <0.001 | 4.13 (2.65 - 6.44) | <0.001 | - |  |
| **Abdominal Obesity (South Asian Cut-off)** |  |  |  |  |  |  |  |  |  |  |
| No | Ref |  | Ref |  | Ref |  | Ref |  | Ref |  |
| Yes | 3.45 (2.48-4.78) | <0.001 | 3.00 (2.08-4.32) | 0.00 | - |  | 1.87 (1.28 - 2.74) | 0.00 | - |  |
| **Abdominal obesity**  **(South Asian Cut-off), body mass index** |  |  |  |  |  |  |  |  |  |  |
| No, Normal | Ref |  | Ref |  | Ref |  | Ref |  | Ref |  |
| No, Underweight | 0.39 (0.17 - 0.91) | 0.03 | - |  | - |  | - |  | 0.39 (0.18 - 0.83) | 0.02 |
| No , Overweight | 1.77 (0.81 - 3.85) | 0.15 | - |  | - |  | - |  | 1.67 (0.73 - 3.82) | 0.22 |
| No, Obesity | 4.92 (1.91 - 12.70) | <0.001 | - |  | - |  | - |  | 3.58 (1.33 - 9.61) | 0.01 |
| Yes, Normal | 1.83 (1.07 - 3.14) | 0.03 | - |  | - |  | - |  | 1.74 (1.00 - 3.04) | 0.05 |
| Yes, Underweight | 1.53 (0.47 - 4.99) | 0.48 | - |  | - |  | - |  | 1.35 (0.42 - 4.39) | 0.61 |
| Yes, Overweight | 3.09 (1.86 - 5.14) | <0.001 | - |  | - |  | - |  | 2.72 (1.57 - 4.73) | <0.001 |
| Yes, Obesity | 9.23 (5.63 - 15.14) | <0.001 | - |  | - |  | - |  | 7.35 (4.34 - 12.47) | <0.001 |

**Table 12: Findings from the logistic regression models showing factors associated with hypertension in Bangladesh**

| **Variables** | **Crude Model** | | **Model 1** | | **Model 2** | | **Model 3** | | **Model 4** | |
| --- | --- | --- | --- | --- | --- | --- | --- | --- | --- | --- |
|  | **OR (95% CI)** | **p-value** | **OR (95% CI)** | **p-value** | **OR (95% CI)** | **p-value** | **OR (95% CI)** | **p-value** | **OR (95% CI)** | **p-value** |
| **Age group** |  |  |  |  |  |  |  |  |  |  |
| 18-29 | Ref |  | Ref |  | Ref |  | Ref |  | Ref |  |
| 30-49 | 2.83 (2.21-3.64) | <0.001 | 2.57 (1.94 - 3.40) | <0.001 | 2.67 (2.01-3.53) | <0.001 | 2.50 (1.88 - 3.31) | <0.001 | 2.50 (1.88 - 3.32) | <0.001 |
| 50-69 | 5.82 (4.54-7.48) | <0.001 | 6.06 (4.57 - 8.02) | <0.001 | 6.74 (5.03-9.04) | <0.001 | 6.19 (4.64 - 8.25) | <0.001 | 6.24 (4.67 - 8.33) | <0.001 |
| **Gender** |  |  |  |  |  |  |  |  |  |  |
| Male | Ref |  | Ref |  | Ref |  | Ref |  | Ref |  |
| Female | 1.66 (1.40-1.97) | <0.001 | 1.29 (1.04 - 1.60) | 0.02 | 1.30 (1.04-1.63) | 0.02 | 1.25 (1.01 - 1.56) | 0.05 | 1.25 (1.00 - 1.57) | 0.05 |
| **Education** |  |  |  |  |  |  |  |  |  |  |
| No formal schooling | Ref |  | Ref |  | Ref |  | Ref |  | Ref |  |
| Up to primary | 0.77 (0.63-0.94) | 0.01 | 1.14 (0.93 - 1.41) | 0.20 | 1.18 (0.96-1.45) | 0.11 | 1.12 (0.91 - 1.37) | 0.29 | 1.10 (0.90 - 1.35) | 0.36 |
| Up to secondary | 0.79 (0.62-1.02) | 0.07 | 1.39 (1.05 - 1.85) | 0.02 | 1.42 (1.08-1.88) | 0.01 | 1.32 (1.00 - 1.76) | 0.05 | 1.30 (0.98 - 1.73) | 0.07 |
| College and higher | 0.79 (0.54-1.15) | 0.22 | 0.99 (0.68 - 1.44) | 0.95 | 1.11 (0.74-1.65) | 0.61 | 0.97 (0.66 - 1.42) | 0.86 | 0.95 (0.64 - 1.39) | 0.78 |
| **Current smoking** |  |  |  |  |  |  |  |  |  |  |
| Yes | Ref |  | Ref |  | Ref |  | Ref |  | Ref |  |
| No | 1.91 (1.46-2.49) | <0.001 | 1.65 (1.21 - 2.25) | 0.00 | 1.54 (1.11-2.14) | 0.01 | 1.55 (1.12 - 2.13) | 0.01 | 1.58 (1.15 - 2.16) | 0.01 |
| **Alcohol use in last 30 days** |  |  |  |  |  |  |  |  |  |  |
| Yes | Ref |  | Ref |  | Ref |  | Ref |  | Ref |  |
| No | 1.86 (1.24-2.78) | <0.001 | 0.96 (0.61 - 1.51) | 0.86 | 0.98 (0.62-1.55) | 0.94 | 0.99 (0.63 - 1.56) | 0.97 | 0.96 (0.61 - 1.51) | 0.86 |
| **Sufficient fruit and vegetable intake** |  |  |  |  |  |  |  |  |  |  |
| Yes | Ref |  | Ref |  | Ref |  | Ref |  | Ref |  |
| No | 1.23 (1.00-1.51) | 0.05 | 1.24 (0.98 - 1.56) | 0.07 | 1.20 (0.95-1.52) | 0.13 | 1.25 (0.98 - 1.57) | 0.07 | 1.25 (0.99 - 1.58) | 0.06 |
| **Adequate physical activity** |  |  |  |  |  |  |  |  |  |  |
| Yes | Ref |  | Ref |  | Ref |  | Ref |  | Ref |  |
| No | 1.81 (1.41-2.33) | <0.001 | 1.21 (0.93 - 1.57) | 0.16 | 1.24 (0.95-1.61) | 0.11 | 1.21 (0.93 - 1.58) | 0.15 | 1.21 (0.93 - 1.58) | 0.15 |
| **Diabetes** |  |  |  |  |  |  |  |  |  |  |
| No | Ref |  | Ref |  | Ref |  | Ref |  | Ref |  |
| Yes | 4.11 (3.02-5.59) | <0.001 | 2.37 (1.75 - 3.21) | 0.00 | 2.29 (1.68-3.11) | <0.001 | 2.21 (1.62 - 3.00) | <0.001 | 2.22 (1.63 - 3.02) | <0.001 |
| **BMI category** |  |  |  |  |  |  |  |  |  |  |
| Normal Weight | Ref |  | Ref |  | Ref |  | Ref |  | Ref |  |
| Under weight | 0.54 (0.39 - 0.75) | <0.001 | - |  | 0.50 (0.36-0.70) | <0.001 | 0.68 (0.47 - 0.97) | 0.04 | - |  |
| Overweight | 2.21 (1.79 - 2.73) | <0.001 | - |  | 1.95 (1.55-2.46) | <0.001 | 1.38 (1.09 - 1.76) | 0.01 | - |  |
| Obesity | 3.42 (2.61 - 4.49) | <0.001 | - |  | 2.61 (1.99-3.43) | <0.001 | 1.80 (1.35 - 2.39) | <0.001 | - |  |
| **Abdominal Obesity (South Asian Cut-off)** |  |  |  |  |  |  |  |  |  |  |
| No | Ref |  | Ref |  | Ref |  | Ref |  | Ref |  |
| Yes | 3.98 (3.22-4.90) | <0.001 | 3.20 (2.49-4.11) | <0.001 | - |  | 2.25 (1.69 - 3.00) | <0.001 | - |  |
| **Abdominal obesity**  **(South Asian Cut-off), body mass index** |  |  |  |  |  |  |  |  |  |  |
| No, Normal | Ref |  | Ref |  | Ref |  | Ref |  | Ref |  |
| No, Underweight | 0.97 (0.67 - 1.41) | 0.89 | - |  | - |  | - |  | 0.87 (0.60 - 1.27) | 0.47 |
| No , Overweight | 1.98 (1.10 - 3.56) | 0.02 | - |  | - |  | - |  | 2.22 (1.21 - 4.07) | 0.01 |
| No, Obesity | 2.20 (0.51 - 9.46) | 0.29 | - |  | - |  | - |  | 2.38 (0.60 - 9.40) | 0.22 |
| Yes, Normal | 3.20 (2.33 - 4.40) | <0.001 | - |  | - |  | - |  | 2.73 (1.94 - 3.84) | <0.001 |
| Yes, Underweight | 0.89 (0.43 - 1.87) | 0.77 | - |  | - |  | - |  | 0.52 (0.22 - 1.20) | 0.12 |
| Yes, Overweight | 4.14 (3.10 - 5.54) | <0.001 | - |  | - |  | - |  | 3.42 (2.43 - 4.81) | <0.001 |
| Yes, Obesity | 6.20 (4.54 - 8.46) | <0.001 | - |  | - |  | - |  | 4.50 (3.17 - 6.40) | <0.001 |

**Table 13: Findings from the logistic regression models showing factors associated with hypertension in Bhutan**

| **Variables** | **Crude Model** | | **Model 1** | | **Model 2** | | **Model 3** | | **Model 4** | |
| --- | --- | --- | --- | --- | --- | --- | --- | --- | --- | --- |
|  | **OR (95% CI)** | **p-value** | **OR (95% CI)** | **p-value** | **OR (95% CI)** | **p-value** | **OR (95% CI)** | **p-value** | **OR (95% CI)** | **p-value** |
| **Age group** |  |  |  |  |  |  |  |  |  |  |
| 18-29 | Ref |  | Ref |  | Ref |  | Ref |  | Ref |  |
| 30-49 | 4.25 (3.30-5.47) | <0.001 | 2.98 (2.17 - 4.11) | <0.001 | 2.93 (2.11-4.08) | <0.001 | 2.76 (1.98 - 3.84) | <0.001 | 2.75 (1.97 - 3.84) | <0.001 |
| 50-69 | 8.78 (6.23-12.39) | <0.001 | 6.16 (4.03 - 9.40) | <0.001 | 6.33 (4.19-9.58) | <0.001 | 5.95 (3.92 - 9.03) | <0.001 | 5.95 (3.90 - 9.08) | <0.001 |
| **Gender** |  |  |  |  |  |  |  |  |  |  |
| Male | Ref |  | Ref |  | Ref |  | Ref |  | Ref |  |
| Female | 0.65 (0.54-0.78) | <0.001 | 0.61 (0.50 - 0.74) | <0.001 | 0.59 (0.48-0.73) | <0.001 | 0.57 (0.47 - 0.70) | <0.001 | 0.57 (0.47 - 0.71) | <0.001 |
| **Education** |  |  |  |  |  |  |  |  |  |  |
| No formal schooling | Ref |  | Ref |  | Ref |  | Ref |  | Ref |  |
| Up to primary | 0.81 (0.63-1.04) | 0.10 | 1.02 (0.75 - 1.40) | 0.89 | 1.00 (0.72-1.37) | 0.98 | 0.99 (0.72 - 1.37) | 0.95 | 0.98 (0.71 - 1.35) | 0.91 |
| Up to secondary | 0.40 (0.32-0.50) | <0.001 | 0.84 (0.64 - 1.10) | 0.21 | 0.82 (0.63-1.08) | 0.15 | 0.81 (0.62 - 1.07) | 0.14 | 0.81 (0.61 - 1.06) | 0.13 |
| College and higher | 0.86 (0.69-1.09) | 0.21 | 0.97 (0.73 - 1.27) | 0.81 | 1.00 (0.76-1.31) | 1.00 | 0.95 (0.73 - 1.25) | 0.73 | 0.96 (0.73 - 1.25) | 0.74 |
| **Current smoking** |  |  |  |  |  |  |  |  |  |  |
| Yes | Ref |  | Ref |  | Ref |  | Ref |  | Ref |  |
| No | 1.48 (1.09-2.00) | 0.01 | 1.20 (0.88 - 1.64) | 0.24 | 1.18 (0.86-1.64) | 0.30 | 1.18 (0.85 - 1.63) | 0.32 | 1.18 (0.85 - 1.63) | 0.31 |
| **Alcohol use in last 30 days** |  |  |  |  |  |  |  |  |  |  |
| Yes | Ref |  | Ref |  | Ref |  | Ref |  | Ref |  |
| No | 0.56 (0.45-0.68) | <0.001 | 0.64 (0.52 - 0.79) | <0.001 | 0.65 (0.53-0.82) | <0.001 | 0.66 (0.53 - 0.82) | <0.001 | 0.66 (0.53 - 0.82) | <0.001 |
| **Sufficient fruit and vegetable intake** |  |  |  |  |  |  |  |  |  |  |
| Yes | Ref |  | Ref |  | Ref |  | Ref |  | Ref |  |
| No | 1.11 (0.94-1.32) | 0.22 | 1.09 (0.91 - 1.30) | 0.36 | 1.12 (0.93-1.34) | 0.22 | 1.11 (0.93 - 1.33) | 0.24 | 1.11 (0.93 - 1.33) | 0.24 |
| **Adequate physical activity** |  |  |  |  |  |  |  |  |  |  |
| Yes | Ref |  | Ref |  | Ref |  | Ref |  | Ref |  |
| No | 1.30 (1.04-1.63) | 0.02 | 1.13 (0.90 - 1.42) | 0.30 | 1.11 (0.88-1.40) | 0.38 | 1.13 (0.89 - 1.42) | 0.31 | 1.13 (0.90 - 1.43) | 0.30 |
| **Diabetes** |  |  |  |  |  |  |  |  |  |  |
| No | Ref |  | Ref |  | Ref |  | Ref |  | Ref |  |
| Yes | 3.76 (2.31-6.13) | <0.001 | 2.09 (1.21 - 3.59) | 0.01 | 1.90 (1.11-3.25) | 0.02 | 1.87 (1.10 - 3.20) | 0.02 | 1.86 (1.09 - 3.17) | 0.02 |
| **BMI category** |  |  |  |  |  |  |  |  |  |  |
| Normal Weight | Ref |  | Ref |  | Ref |  | Ref |  | Ref |  |
| Under weight | 0.42 (0.23-0.74) | <0.001 | - |  | 0.55 (0.31-0.96) | 0.04 | 0.65 (0.37 - 1.16) | 0.14 | - |  |
| Over weight/Obesity | 1.94 (1.51-2.48) | <0.001 | - |  | 1.72 (1.32-2.25) | <0.001 | 1.35 (1.02 - 1.77) | 0.03 | - |  |
| Obesity | 3.47 (2.68-4.48) | <0.001 | - |  | 2.86 (2.14-3.82) | <0.001 | 2.17 (1.59 - 2.96) | <0.001 | - |  |
| **Abdominal Obesity (South Asian Cut-off)** |  |  |  |  |  |  |  |  |  |  |
| No | Ref |  | Ref |  | Ref |  | Ref |  | Ref |  |
| Yes | 3.28 (2.55 - 4.24) | <0.001 | 2.52 (1.92 - 3.31) | 0.00 | - |  | 1.74 (1.31 - 2.32) | <0.001 | - |  |
| **Abdominal obesity**  **(South Asian Cut-off), body mass index** |  |  |  |  |  |  |  |  |  |  |
| No, Normal | Ref |  | Ref |  | Ref |  | Ref |  | Ref |  |
| No, Underweight | 0.41 (0.20 - 0.83) | 0.014 | - |  | - |  | - |  | 0.53 (0.27 - 1.04) | 0.06 |
| No , Overweight | 1.08 (0.59 - 1.99) | 0.795 | - |  | - |  | - |  | 1.04 (0.56 - 1.95) | 0.90 |
| No, Obesity | 0.59 (0.12 - 2.99) | 0.523 | - |  | - |  | - |  | 0.46 (0.10 - 2.19) | 0.33 |
| Yes, Normal | 1.82 (1.25 - 2.65) | 0.002 | - |  | - |  | - |  | 1.48 (1.01 - 2.18) | 0.05 |
| Yes, Underweight | 2.17 (0.76 - 6.20) | 0.148 | - |  | - |  | - |  | 1.70 (0.64 - 4.47) | 0.28 |
| Yes, Overweight | 2.71 (1.97 - 3.73) | <0.001 | - |  | - |  | - |  | 2.20 (1.54 - 3.15) | <0.001 |
| Yes, Obesity | 4.56 (3.34 - 6.23) | <0.001 | - |  | - |  | - |  | 3.54 (2.47 - 5.07) | <0.001 |

**Table 14: Findings from the logistic regression models showing factors associated with hypertension in Nepal**

| **Variables** | **Crude Model** | | **Model 1** | | **Model 2** | | **Model 3** | | **Model 4** | |
| --- | --- | --- | --- | --- | --- | --- | --- | --- | --- | --- |
|  | **OR (95% CI)** | **p-value** | **OR (95% CI)** | **p-value** | **OR (95% CI)** | **p-value** | **OR (95% CI)** | **p-value** | **OR (95% CI)** | **p-value** |
| **Age group** |  |  |  |  |  |  |  |  |  |  |
| 18-29 | Ref |  | Ref |  | Ref |  | Ref |  | Ref |  |
| 30-49 | 2.24 (1.70-2.94) | <0.001 | 1.76 (1.33 - 2.33) | <0.001 | 1.70 (1.27-2.28) | <0.001 | 1.63 (1.22 - 2.18) | <0.001 | 1.64 (1.22 - 2.19) | <0.001 |
| 50-69 | 4.81 (3.65-6.35) | <0.001 | 3.60 (2.54 - 5.11) | <0.001 | 3.80 (2.65-5.44) | <0.001 | 3.63 (2.54 - 5.20) | <0.001 | 3.65 (2.55 - 5.21) | <0.001 |
| **Gender** |  |  |  |  |  |  |  |  |  |  |
| Male | Ref |  | Ref |  | Ref |  | Ref |  | Ref |  |
| Female | 0.58 (0.48-0.71) | <0.001 | 0.56 (0.43 - 0.73) | <0.001 | 0.59 (0.46-0.77) | <0.001 | 0.57 (0.43 - 0.74) | <0.001 | 0.57 (0.44 - 0.74) | <0.001 |
| **Education** |  |  |  |  |  |  |  |  |  |  |
| No formal schooling | Ref |  | Ref |  | Ref |  | Ref |  | Ref |  |
| Up to primary | 0.83 (0.68-1.02) | 0.07 | 0.98 (0.77 - 1.25) | 0.86 | 0.95 (0.73-1.22) | 0.67 | 0.95 (0.73 - 1.22) | 0.66 | 0.95 (0.74 - 1.22) | 0.69 |
| Up to secondary | 0.47 (0.36-0.63) | <0.001 | 0.72 (0.51 - 1.01) | 0.06 | 0.68 (0.48-0.96) | 0.03 | 0.67 (0.48 - 0.95) | 0.02 | 0.68 (0.48 - 0.95) | 0.02 |
| College and higher | 0.66 (0.35-1.25) | 0.20 | 0.88 (0.43 - 1.79) | 0.72 | 0.78 (0.41-1.49) | 0.45 | 0.78 (0.40 - 1.51) | 0.46 | 0.78 (0.42 - 1.46) | 0.44 |
| **Current smoking** |  |  |  |  |  |  |  |  |  |  |
| Yes | Ref |  | Ref |  | Ref |  | Ref |  | Ref |  |
| No | 0.70 (0.56-0.89) | <0.001 | 1.16 (0.90 - 1.48) | 0.25 | 1.15 (0.89-1.49) | 0.29 | 1.13 (0.88 - 1.46) | 0.33 | 1.13 (0.88 - 1.46) | 0.35 |
| **Alcohol use in last 30 days** |  |  |  |  |  |  |  |  |  |  |
| Yes | Ref |  | Ref |  | Ref |  | Ref |  | Ref |  |
| No | 0.51 (0.41-0.63) | <0.001 | 0.64 (0.50 - 0.82) | <0.001 | 0.63 (0.48-0.81) | <0.001 | 0.64 (0.49 - 0.83) | <0.001 | 0.64 (0.49 - 0.83) | <0.001 |
| **Sufficient fruit and vegetable intake** |  |  |  |  |  |  |  |  |  |  |
| Yes | Ref |  | Ref |  | Ref |  | Ref |  | Ref |  |
| No | 1.26 (0.88-1.81) | 0.20 | 1.25 (0.90 - 1.73) | 0.19 | 1.23 (0.89-1.71) | 0.21 | 1.24 (0.90 - 1.71) | 0.19 | 1.23 (0.89 - 1.71) | 0.21 |
| **Adequate physical activity** |  |  |  |  |  |  |  |  |  |  |
| Yes | Ref |  | Ref |  | Ref |  | Ref |  | Ref |  |
| No | 1.00 (0.75-1.35) | 0.98 | 0.95 (0.70 - 1.29) | 0.74 | 0.92 (0.68-1.24) | 0.58 | 0.91 (0.68 - 1.23) | 0.54 | 0.91 (0.67 - 1.22) | 0.52 |
| **Diabetes** |  |  |  |  |  |  |  |  |  |  |
| No | Ref |  | Ref |  | Ref |  | Ref |  | Ref |  |
| Yes | 2.40 (1.69-3.42) | <0.001 | 1.87 (1.37 - 2.57) | <0.001 | 1.77 (1.28-2.44) | <0.001 | 1.77 (1.29 - 2.42) | <0.001 | 1.75 (1.28 - 2.40) | <0.001 |
| **BMI category** |  |  |  |  |  |  |  |  | blank cell |  |
| Normal Weight | Ref |  | Ref |  | Ref |  | Ref |  | Ref |  |
| Under weight | 0.71 (0.47-1.09) | 0.12 | - |  | 0.67 (0.43-1.05) | 0.08 | 0.75 (0.49 - 1.15) | 0.19 | - |  |
| Over weight/Obesity | 1.54 (1.21-1.95) | <0.001 | - |  | 1.53 (1.17-1.99) | <0.001 | 1.43 (1.08 - 1.90) | 0.01 | - |  |
| Obesity | 3.18 (2.40-4.22) | <0.001 | - |  | 3.24 (2.37-4.43) | <0.001 | 2.98 (2.15 - 4.14) | <0.001 | - |  |
| **Abdominal Obesity (South Asian Cut-off)** |  |  |  |  |  |  |  |  |  |  |
| No | Ref |  | Ref |  | Ref |  | Ref |  | Ref |  |
| Yes | 1.852 (1.447 - 2.371) | <0.001 | 1.83 (1.40 - 2.38) | <0.001 | - |  | 1.35 (1.02 - 1.79) | 0.04 | - |  |
| **Abdominal obesity**  **(South Asian Cut-off), body mass index** |  |  |  |  |  |  |  |  |  |  |
| No, Normal | Ref |  | Ref |  | Ref |  | Ref |  | Ref |  |
| No, Underweight | 0.917 (0.532 - 1.581) | 0.755 | - |  | - |  | - |  | 0.84 (0.47 - 1.51) | 0.55 |
| No , Overweight | 1.800 (0.789 - 4.106) | 0.162 | - |  | - |  | - |  | 1.77 (0.73 - 4.30) | 0.21 |
| No, Obesity | 3.247 (0.987 - 10.681) | 0.053 | - |  | - |  | - |  | 3.43 (0.89 - 13.28) | 0.07 |
| Yes, Normal | 1.554 (1.086 - 2.223) | 0.016 | - |  | - |  | - |  | 1.50 (1.03 - 2.19) | 0.04 |
| Yes, Underweight | 1.035 (0.522 - 2.053) | 0.921 | - |  | - |  | - |  | 0.98 (0.46 - 2.10) | 0.97 |
| Yes, Overweight | 2.076 (1.496 - 2.880) | <0.001 | - |  | - |  | - |  | 2.03 (1.43 - 2.88) | <0.001 |
| Yes, Obesity | 4.291 (2.895 - 6.361) | <0.001 | - |  | - |  | - |  | 4.30 (2.81 - 6.58) | <0.001 |

**Table 15: Findings from the logistic regression models showing factors associated with hypertension in Sri Lanka**

| **Variables** | **Crude Model** | | **Model 1** | | **Model 2** | | **Model 3** | | **Model 4** | |
| --- | --- | --- | --- | --- | --- | --- | --- | --- | --- | --- |
|  | **OR (95% CI)** | **p-value** | **OR (95% CI)** | **p-value** | **OR (95% CI)** | **p-value** | **OR (95% CI)** | **p-value** | **OR (95% CI)** | **p-value** |
| **Age group** |  |  |  |  |  |  |  |  |  |  |
| 18-29 | Ref |  | Ref |  | Ref |  | Ref |  | Ref |  |
| 30-49 | 2.97 (2.10-4.20) | <0.001 | 2.51 (1.76 - 3.57) | <0.001 | 2.30 (1.61-3.29) | <0.001 | 2.26 (1.58 - 3.23) | <0.001 | 2.27 (1.59 - 3.25) | <0.001 |
| 50-69 | 9.75 (6.91-13.76) | <0.001 | 7.97 (5.59 - 11.37) | <0.001 | 7.80 (5.46-11.13) | <0.001 | 7.59 (5.31 - 10.84) | <0.001 | 7.66 (5.36 - 10.95) | <0.001 |
| **Gender** |  |  |  |  |  |  |  |  |  |  |
| Male | Ref |  | Ref |  | Ref |  | Ref |  | Ref |  |
| Female | 1.14 (0.97-1.35) | 0.12 | 1.01 (0.79 - 1.31) | 0.92 | 1.01 (0.78-1.30) | 0.97 | 0.96 (0.74 - 1.24) | 0.76 | 0.96 (0.74 - 1.25) | 0.77 |
| **Education** |  |  |  |  |  |  |  |  |  |  |
| No formal schooling | Ref |  | Ref |  | Ref |  | Ref |  | Ref |  |
| Up to primary | 0.78 (0.53-1.15) | 0.21 | 0.71 (0.48 - 1.05) | 0.08 | 0.75 (0.50-1.12) | 0.15 | 0.74 (0.50 - 1.10) | 0.14 | 0.74 (0.50 - 1.11) | 0.14 |
| Up to secondary | 0.53 (0.38-0.75) | <0.001 | 0.86 (0.61 - 1.22) | 0.40 | 0.86 (0.60-1.24) | 0.42 | 0.84 (0.58 - 1.21) | 0.34 | 0.84 (0.59 - 1.21) | 0.35 |
| College and higher | 0.42 (0.25-0.71) | <0.001 | 0.69 (0.41 - 1.16) | 0.16 | 0.67 (0.39-1.15) | 0.14 | 0.64 (0.37 - 1.10) | 0.11 | 0.64 (0.38 - 1.11) | 0.11 |
| **Current smoking** |  |  |  |  |  |  |  |  |  |  |
| Yes | Ref |  | Ref |  | Ref |  | Ref |  | Ref |  |
| No | 1.10 (0.86-1.41) | 0.46 | 1.07 (0.79 - 1.47) | 0.65 | 1.05 (0.77-1.45) | 0.75 | 1.02 (0.74 - 1.41) | 0.90 | 1.02 (0.74 - 1.40) | 0.92 |
| **Alcohol use in last 30 days** |  |  |  |  |  |  |  |  |  |  |
| Yes | Ref |  | Ref |  | Ref |  | Ref |  | Ref |  |
| No | 0.85 (0.71-1.01) | 0.07 | 0.87 (0.67 - 1.13) | 0.31 | 0.87 (0.67-1.14) | 0.31 | 0.87 (0.67 - 1.14) | 0.32 | 0.88 (0.67 - 1.15) | 0.34 |
| **Sufficient fruit and vegetable intake** |  |  |  |  |  |  |  |  |  |  |
| Yes | Ref |  | Ref |  | Ref |  | Ref |  | Ref |  |
| No | 0.81 (0.68-0.97) | 0.02 | 0.85 (0.70 - 1.02) | 0.08 | 0.85 (0.71-1.03) | 0.10 | 0.86 (0.71 - 1.04) | 0.11 | 0.86 (0.71 - 1.03) | 0.11 |
| **Adequate physical activity** |  |  |  |  |  |  |  |  |  |  |
| Yes | Ref |  | Ref |  | Ref |  | Ref |  | Ref |  |
| No | 1.38 (1.00-1.90) | 0.05 | 1.00 (0.70 - 1.43) | 1.00 | 1.01 (0.70-1.46) | 0.95 | 0.99 (0.69 - 1.43) | 0.97 | 0.99 (0.69 - 1.43) | 0.96 |
| **Diabetes** |  |  |  |  |  |  |  |  |  |  |
| No | Ref |  | Ref |  | Ref |  | Ref |  | Ref |  |
| Yes | 3.53 (2.80-4.45) | <0.001 | 2.03 (1.61 - 2.56) | <0.001 | 1.98 (1.57-2.50) | <0.001 | 1.94 (1.53 - 2.44) | <0.001 | 1.94 (1.54 - 2.46) | <0.001 |
| **BMI category** |  |  |  |  |  |  |  |  |  |  |
| Normal Weight | Ref |  | Ref |  | Ref |  | Ref |  | Ref |  |
| Under weight | 0.85 (0.65-1.13) | 0.27 | - |  | 0.97 (0.72-1.31) | 0.86 | 1.21 (0.89 - 1.65) | 0.23 | - |  |
| Over weight/Obesity | 1.99 (1.64-2.41) | <0.001 | - |  | 1.80 (1.47-2.20) | <0.001 | 1.60 (1.31 - 1.96) | <0.001 | - |  |
| Obesity | 3.72 (2.99-4.63) | <0.001 | - |  | 3.31 (2.56-4.29) | <0.001 | 2.96 (2.28 - 3.83) | <0.001 | - |  |
| **Abdominal Obesity (South Asian Cut-off)** |  |  |  |  |  |  |  |  |  |  |
| No | Ref |  | Ref |  | Ref |  | Ref |  | Ref |  |
| Yes | 2.71 (2.17 - 3.40) | <0.001 | 2.14 (1.67-2.74) | <0.001 | - |  | 1.67 (1.28 - 2.17) | <0.001 | - |  |
| **Abdominal obesity**  **(South Asian Cut-off), body mass index** |  |  |  |  |  |  |  |  |  |  |
| No, Normal | Ref |  | Ref |  | Ref |  | Ref |  | Ref |  |
| No, Underweight | 1.13 (0.74 - 1.72) | 0.568 | - |  | - |  | - |  | 1.16 (0.74 - 1.82) | 0.52 |
| No , Overweight | 1.47 (0.75 - 2.89) | 0.26 | - |  | - |  | - |  | 0.98 (0.50 - 1.91) | 0.95 |
| No, Obesity | 2.61 (0.89 - 7.62) | 0.079 | - |  | - |  | - |  | 2.27 (0.72 - 7.12) | 0.16 |
| Yes, Normal | 1.88 (1.35 - 2.61) | <0.001 | - |  | - |  | - |  | 1.52 (1.07 - 2.17) | 0.02 |
| Yes, Underweight | 2.10 (1.30 - 3.39) | 0.003 | - |  | - |  | - |  | 1.79 (1.10 - 2.91) | 0.02 |
| Yes, Overweight | 3.18 (2.30 - 4.41) | <0.001 | - |  | - |  | - |  | 2.54 (1.79 - 3.61) | <0.001 |
| Yes, Obesity | 5.93 (4.24 - 8.30) | <0.001 | - |  | - |  | - |  | 4.65 (3.16 - 6.82) | <0.001 |
